# Impact of administration routes and dose frequency on the toxicology of SARS-CoV-2 mRNA vaccines in mice model

**DOI:** 10.1007/s00204-024-03912-1

**Published:** 2024-12-10

**Authors:** Jae-Hun Ahn, Jisun Lee, Gahyun Roh, Na-Young Lee, Hee-Jin Bae, Euna Kwon, Kang-Min Han, Ji-Eun Kim, Hyo-Jung Park, Soyeon Yoo, Sung Pil Kwon, Eun-Kyoung Bang, Gyochang Keum, Jae-Hwan Nam, Byeong-Cheol Kang

**Affiliations:** 1 Department of Experiment Animal Research, Biomedical Research Institute, Seoul National University Hospital, Seoul, Republic of Korea; 2 Graduate School of Translational Medicine, Seoul National University College of Medicine, Seoul, Republic of Korea; 3 Department of Veterinary Pathology and Research Institute of Veterinary Science, College of Veterinary Medicine, Seoul National University, Seoul, Republic of Korea; 4 Department of Medical and Biological Sciences, The Catholic University of Korea, Bucheon, Republic of Korea; 5 BK Four Department of Biotechnology, The Catholic University of Korea, Bucheon, Republic of Korea; 6 Center for Brain Technology, Brain Science Institute, Korea Institute of Science and Technology, Seoul, Republic of Korea; 7 Department of Pathology, CHA Ilsan Medical Center, CHA University, Goyang-si, Republic of Korea

**Keywords:** mRNA vaccine, Lipid nanoparticle, Toxicity, Safety, SARS-CoV-2

## Abstract

The increasing use of SARS-CoV-2 mRNA vaccines has raised concerns about their potential toxicological effects, necessitating further investigation to ensure their safety. To address this issue, we aimed to evaluate the toxicological effects of SARS-CoV-2 mRNA vaccine candidates formulated with four different types of lipid nanoparticles in ICR mice, focusing on repeated doses and administration routes. We conducted an extensive analysis in which mice received the mRNA vaccine candidates intramuscularly (50 μg/head) twice at 2-week intervals, followed by necropsy at 2 and 14 dpsi (days post-secondary injection). In addition, we performed a repeated dose toxicity test involving three, four, or five doses and compared the toxicological outcomes between intravenous and intramuscular routes. Our findings revealed that all vaccine candidates significantly induced SARS-CoV-2 spike protein-specific IgG and T cell responses. However, at 2 dpsi, there was a notable temporary decrease in lymphocyte and reticulocyte counts, anemia-related parameters, and significant increases in cardiac damage markers, troponin-I and NT-proBNP. Histopathological analysis revealed severe inflammation and necrosis at the injection site, decreased erythroid cells in bone marrow, cortical atrophy of the thymus, and increased spleen cellularity. While most toxicological changes observed at 2 dpsi had resolved by 14 dpsi, spleen enlargement and injection site damage persisted. Furthermore, repeated doses led to the accumulation of toxicity, and different administration routes resulted in distinct toxicological phenotypes. These findings highlight the potential toxicological risks associated with mRNA vaccines, emphasizing the necessity to carefully consider administration routes and dosage regimens in vaccine safety evaluations, particularly given the presence of bone marrow and immune organ toxicity, which, though eventually reversible, remains a serious concern.

**Supplementary Information:**

The online version contains supplementary material available at 10.1007/s00204-024-03912-1.

## Introduction

Messenger RNA (mRNA) vaccines consist of target protein-coding mRNA and lipid nanoparticles (LNP). mRNA is unstable and induces strong inflammatory responses by activating pattern recognition receptors in host cells. Consequently, scientists have conducted extensive trials to enhance mRNA’s stability and reduce inflammation, resulting in the development of several novel mRNA platforms (Lundstrom 2020; Thess et al. 2015; Wang and Wang 2015; Weng et al. 2020). Nucleoside-modified mRNA (modRNA), a key platform, substitutes uracil with pseudouracil to boost stability and reduce receptor activity. This platform has been utilized in BNT162b2 and mRNA-1273, the first mRNA vaccines approved for SARS-CoV-2 (Granados-Riveron and Aquino-Jarquin 2021; Pardi and Weissman 2017). Typically, LNP comprises ionizable lipid, helper lipid, cholesterol, and polyethylene glycol (PEG), with the precise combination of these components critically determining the delivery and efficacy of mRNA expression (Hou et al. 2021). Thus, various LNPs have been developed to optimize mRNA delivery and expression. The advancement of such sophisticated technology has led to the commercialization of mRNA vaccines, expanding their application from infectious disease prevention to therapeutic treatments for cancer and autoimmune diseases (Krienke et al. 2021; Miao et al. 2021).

The SARS-CoV-2 pandemic necessitated the rapid authorization of mRNA vaccines for human use (Oliver et al. 2020), termed emergency use authorization (EUA). This expedited approval was driven not only by the urgent nature of the pandemic but also by the lack of pre-clinical toxicity testing guidelines for mRNA vaccines. Consequently, the World Health Organization (WHO) promptly issued a document, ‘Evaluation of the quality, safety and efficacy of messenger RNA vaccines for the prevention of infectious diseases: regulatory considerations’ (*WHO. Evaluation of the quality, safety and efficacy of messenger RNA vaccines for the prevention of infectious diseases: regulatory considerations, Technical document, Annex 3*, 2021). Assessing the potential toxicity of modRNA and novel composite LNPs was highlighted as crucial in the guideline. However, the WHO guideline has several limitations. Since there had been only two approved mRNA vaccines (BNT162b2 and mRNA-1273), there was little accumulated data regarding the pre-clinical safety tests of mRNA vaccines. Therefore, specific technical guidance for mRNA vaccine safety tests such as route of administration, repeated dose, and pivotal biomarkers for specific toxicity are needed to establish accurate and reliable pre-clinical toxicity test guidelines.

Numerous studies have focused on the efficacy of modRNA formulated with novel LNPs, while research on their safety remains limited (El Sahly et al. 2021; Elia et al. 2021; Huang et al. 2021; Kremsner et al. 2022; Laczkó et al. 2020; Ying et al. 2021). According to the WHO guidelines on pre-clinical vaccine evaluation, even if a vaccine is intended for intramuscular administration during clinical trials, it is advisable to conduct toxicity testing through both intramuscular and intravenous routes. This approach facilitates a comprehensive understanding of the vaccine’s potential risks and toxicity spectrum (WHO 2005, 2013). However, in the EUA processes for SARS-CoV-2 mRNA vaccines, toxicity assessment was limited to the intramuscular route, and no comparative studies on administration routes have been reported (FDA 2022a, b). Moreover, while SARS-CoV-2 mRNA vaccines have been administered in more than two doses clinically, only two repeated administrations were evaluated in the pre-clinical toxicity assessments for these vaccines, and the risks associated with repeated mRNA vaccine administration have not yet been identified.

This study aims to elucidate the potential toxicity of mRNA vaccines during the acute and recovery phases and to comprehensively assess the toxicological phenotypes of four different mRNA vaccine types based on administration route and repeated dose.

## Materials and methods

### Preparation of mRNA

The antigen was designed using a DNA sequence that encodes the spike protein of the SARS-CoV-2 Omicron variant. The mRNA vaccine plasmid was constructed by integrating antigen DNA into multiple cloning sites on the mRNA platform using restriction enzymes Pac1 and Cla1 (Park et al. 2023). The mRNA vaccination template was linearized with Not1 and then transcribed using the EZ T7 High Yield In vitro Transcription Kit (Enzynomics, Daejeon, Korea), following the manufacturer’s instructions. The mRNA was capped with SC101 (STPharm, Siheung, Korea), and UTP was replaced with N1-methyl-pseudouridine (Trilink, San Diego, CA, USA). The total mRNA was precipitated with lithium chloride and purified using cellulose, as previously described (Baiersdörfer et al. 2019).

### Formulation and characterization of LNPs for the mRNA vaccine

To elucidate the comprehensive potential toxicity of SARS-CoV-2 mRNA vaccines, we selected candidates deemed potentially effective as vaccines, based on results from in vitro expression tests (data not shown). To improve vaccine efficacy, we encapsulated a methyl pseudo uridine-modified SARS-CoV-2 spike protein-coding mRNA platform (CUK3-1) into four different types of lipid nanoparticles (LNPs) comprising SM-102 or I82 or I98, and 1,2-distearoyl-sn-glycero-3 phosphocholine (DSPC) or 1,2-dioleoyl-sn-glycero-3-phosphoethanolamine (DOPE). SM-102 and DSPC are components of the mRNA-1273, and the functionality of I82 or I98 as ionizable lipids was validated in our pilot studies (data not shown). In addition, several trials have employed DOPE as a helper lipid in mRNA vaccine development (Álvarez-Benedicto et al. 2022; Chen et al. 2022; Ermilova and Swenson 2020; Shimosakai et al. 2022). In contrast, the ionizable lipid C12-200 was chosen as a positive toxic control for this study because its effectiveness for RNA delivery has been confirmed (Hajj et al. 2020; Kauffman et al. 2015; Love et al. 2010), and significant toxicity reports such as increases in interleukin-6, liver necrosis, and pancreatic inflammation have also been documented (Whitehead et al. 2014). Previous studies have demonstrated that administering an mRNA vaccine against SARS-CoV-2 induces the production of IgG antibodies and IFN-γ secretion specific to the spike protein in animal models (Chaudhary et al. 2021; Zhang et al. 2020). Considering these backgrounds, LNP formulations were selected for this study.

SM-102 (Cat #: 2089-251-47-6) was purchased from Hanmi Fine Chemical Co. Ltd. (Gyeonggi-do, Republic of Korea). C12-200 (Cat #: HY-145405) was acquired from MedChemExpress (Monmouth Junction, NJ, United States). 1,2-Dimyristoyl-rac-glycero-3-methoxypolyethylene glycol-2000 (DMG-PEG2K, Cat #: 880151P), 1,2-dioleoyl-sn-glycero-3-phosphoethanolamine (DOPE, Cat #: 850725P), and 1,2‐distearoyl‐sn‐glycero‐3‐phosphocholine (DSPC, Cat #: 850365P) were sourced from Avanti Polar Lipids, Inc. (Alabaster, AL 35007, United States). Cholesterol (Cat #: C3045) was obtained from Sigma-Aldrich (St. Louis, MO, United States). n-Butyl lithocholate and trehalose-6,6'-dioleate (TDO), I98, and I82 were synthesized at the Korea Institute of Science and Technology (Seoul, Republic of Korea).

All lipid components were dissolved in a mixture of chloroform and methanol in equal parts, at a concentration of 50 mg/mL. These lipids included ionizable lipids (SM-102, I82, I98, C12-200), substitute ionizable lipid (TDO), PEGylated lipids (DMG-PEG2K), phospholipids (DOPE and DSPC), and steroids (n-butyl lithocholate and cholesterol). The lipid mixtures were prepared using specific molar ratios as follows: LNP-A (SM-102:TDO:DMG-PEG2K:DOPE: n-butyl lithocholate = 25:25:1.5:10:38.5), LNP-B (I82:DMG-PEG2K:DSPC:cholesterol = 50:1.5:10:38.5), LNP-C (I98:DMG-PEG2K:DSPC:cholesterol = 50:1.5:10:38.5), and LNP-D (C12-200:DMG-PEK2K:DOPE:cholesterol = 35:2.5:16:46.5). After concentration under reduced pressure, the lipid mixtures were re-dissolved in ethanol. The mRNA was dissolved in a pH 4.0 buffer solution containing 50 mM sodium citrate. mRNA/LNPs were formulated using NanoAssemblr^®^ Ignite^™^ with a total flow rate of 10 ml/min. The mixing volume ratio was 1:3 between the organic lipid and aqueous mRNA solutions. The N/P charge ratio of ionizable lipid to mRNA was 3 for LNP-A and 6 for LNPs-B and C, while D maintained a 10:1 w% ratio of ionizable lipid to mRNA (Kauffman et al. 2015). The mRNA/LNP solutions underwent buffer exchange using 1X D-PBS and were concentrated to 1 mg/ml based on mRNA content using an Amicon® Ultra-15 Centrifugal Filter with a LABOGENE 624R Centrifuge.

The size, polydispersity index (PDI), and zeta potential of the formulated LNPs were determined using a Zetasizer Ultra (Malvern Panalytical Ltd.). The mRNA encapsulation efficiency of the LNPs was estimated using a standard RiboGreen assay (R11490, InvitrogenTM).

The patent information for LNP synthesis and physicochemical properties of CUK3-1/LNPs is detailed in Supplementary Data (Supplementary data 1A and B).

### Animal experiment

All animal experiment procedures were approved by the Institutional Animal Care and Use Committee of Seoul National University Hospital (Approval No. 22–0088). Six-week-old CrlOri:CD1(ICR) mice were purchased from Orient Bio Inc (Seongnam, Kyonggi-do, South Korea). The animals were housed in individually ventilated cages under specific pathogen-free (SPF) conditions, with temperatures maintained at 21–25 °C and humidity at 30–70%. Mice were adopted for a week before vaccine administration at 7 weeks of age.

Several studies have conducted toxicological tests for mRNA vaccines in animal experimental models, adopting a protocol of repeated administration at set intervals (Donahue et al. 2023; Sedic et al. 2018; Stokes et al. 2020). This scientific consensus likely stems from the two to five doses repeated administration regimen of SARS-CoV2 mRNA vaccines in clinical practice. Consequently, our experimental design involves two doses repeated administration at 2-week intervals for Study #I and III, and three to five doses repeated administration for Study #II. The administration concentration was determined to maximize toxicity based on LNPs solubility, the dosage of mRNA-1273 in the pre-clinical toxicity test and the maximum volume injectable into muscle.

In Study #I, male and female mice were intramuscularly injected with four types of mRNA vaccine candidates (50 µg/100 µl/head) twice, at 2-week intervals, and necropsy was performed either 2 or 14 days post the second injection.

In Study #II, male and female mice were intramuscularly injected with CUK3-1/LNP-B (50 µg/100 µl/head) with two to five doses at 2-week intervals, and necropsy was carried out 2 days post the final injection.

In Study #III, male mice received either intramuscular or intravenous injections of CUK3-1/LNP-B or CUK3-1/LNP-C (100 µg/100 µl/head) twice at 2-week intervals, and necropsies were conducted 2 days post the second injection.

The evaluations included body weight change, complete blood count, blood chemistry, ELISA, organ weight, and histopathologic analysis.

### Complete blood count (CBC)

Blood collection was conducted under isoflurane anesthesia via the caudal vena cava, and samples were immediately collected into K2 EDTA blood collection tubes (BD Biosciences, Franklin Lakes, NJ, USA). The following CBC parameters were analyzed using the automatic hematology analyzer ADVIA 2120i (Siemens Diagnostics, Tarrytown, NY, USA): leukocytes, red blood cells, hemoglobin, hematocrit, mean corpuscular volume (MCV), mean corpuscular hemoglobin (MCH), mean corpuscular hemoglobin concentration (MCHC), platelets, plateletcrit, differential leukocytes, and reticulocytes.

### Serum biochemistry, cTroponin-I (cTnI), and NT-proBNP measurement

Blood samples were collected in serum-separating tubes (BD biosciences) and centrifuged at 2000 RCF for 15 min. The following serum biochemistry parameters were analyzed using the automatic chemistry analyzer 7070 (Hitachi, Tokyo, Japan): calcium, inorganic phosphorus, glucose, blood urea nitrogen (BUN), creatinine, total cholesterol, total protein, albumin, total bilirubin, alkaline phosphatase (ALP), aspartate transaminase (AST), alanine transaminase (ALT), triglycerides, high-density lipoprotein (HDL) cholesterol, low-density lipoprotein (LDL) cholesterol, Na, K and Cl. Levels of cTnI and NT-proBNP in serum were determined using enzyme-linked immunosorbent assays (ELISA) with mouse cTroponin-I ELISA kit (Cat #: CSB-E08421m, Cusabio, Houston, TX, USA) and mouse NT-proBNP ELISA kit (Cat #:MBS2501591, MyBioSource, San Diego, CA, USA) according to the manufacturer’s instructions.

### Histopathological analysis

After the necropsy, tissues were fixed in 10% neutral formalin for 72 h, followed by tissue processing and paraffin embedding; subsequently, the paraffin blocks were sectioned at 4 μm and stained with H&E. Histopathological examination was conducted under microscopy, and histopathological changes were analyzed by experts specializing in laboratory animal pathology.

### SARS-CoV-2 spike protein-specific IgG measurement (ELISA)

Antigen-specific total IgG levels in mouse serum were assessed via enzyme-linked immunosorbent assay (ELISA). Briefly, a 96-well plate was coated with the S protein from the SARS-CoV-2 Omicron variant at a concentration of 100 ng/well and incubated at 4 °C overnight. The wells were blocked with 100 µl of blocking buffer (1% BSA in PBS) for 1 h at room temperature. Serum samples diluted in blocking buffer (1:20) were added to the wells and incubated for 2 h at room temperature (20–22 °C). Following incubation, the wells were washed three times with 200 µl PBS-T (PBS with Tween 20). Horseradish peroxidase-conjugated anti-mouse IgG antibodies (from Bethyl Laboratories, Montgomery, TX, USA) were added to the wells and incubated at room temperature for 1 h. The antibodies were properly diluted in blocking buffer (1:5000). After three washes with PBS-T, tetramethylbenzidine substrate was added to the wells, and the plates were incubated for 15 min. The reaction was stopped by adding 2 N H2SO4. The optical density was then measured at 450 nm using a microplate reader (GloMax Explorer, Promega, Seoul, Republic of Korea).

### Enzyme-Linked ImmunoSpot (ELISpot) assay

Splenocytes (5 × 10^5^) from mRNA vaccine-injected mice at 14 dpsi of Study #1 were cultured in 96-well MultiScreen-IP Filter Plates (Millipore, Burlington, MA, USA) and stimulated with antigen peptides of the spike protein from the Wuhan SARS-CoV-2 strain (5 μg/ml) in RPMI medium for 24 h at 37 °C. The ELISpot assay for detecting IFN-γ released by splenocytes was conducted in accordance with the manufacturer’s instructions (Mab-tech, Stockholm, Sweden).

### Flow cytometry

Splenocytes (1 × 10^6^) were isolated from mRNA vaccine-injected mice at 14 dpsi of Study #1 and cultured in 96-well plates. The samples were treated with Brefeldin A (Golgi plug, BD Biosciences, Franklin Lakes, NY, USA) and stimulated with antigen peptides of the spike protein from the SARS-CoV-2 Omicron variant (5 μg/mL) in RPMI medium for 12 h at 37 °C. Afterward, the splenocytes were treated with anti-mouse CD16/32 (Invitrogen, Waltham, Massachusetts, USA) for 20 min at 4 °C. Splenocytes were stained with anti-mouse CD4, CD8, CD69, CD25, ICOS, and PD-1 fluorescent antibodies for 30 min at 4 °C in the dark. Samples were analyzed using a CytoFlex flow cytometer (Beckman Coulter, Brea, CA, USA), and the data were processed with CytExpert.

### Statistical analysis

The statistical significance of the differences among groups in Study #I and III was determined using the Kruskal–Wallis test followed by Bonferroni post hoc analysis (SPSS Statistics, Chicago, IN, USA). The statistical significance of the differences between two groups and repeated doses in Study #II was ascertained through two-way ANOVA followed by Bonferroni’s multiple post hoc analyses. *p* values < 0.05 were considered statistically significant.

## Results

### mRNA vaccine candidates efficiently induce the SARS-CoV-2 spike protein-specific IgG and T cell responses

**Fig. 1:**
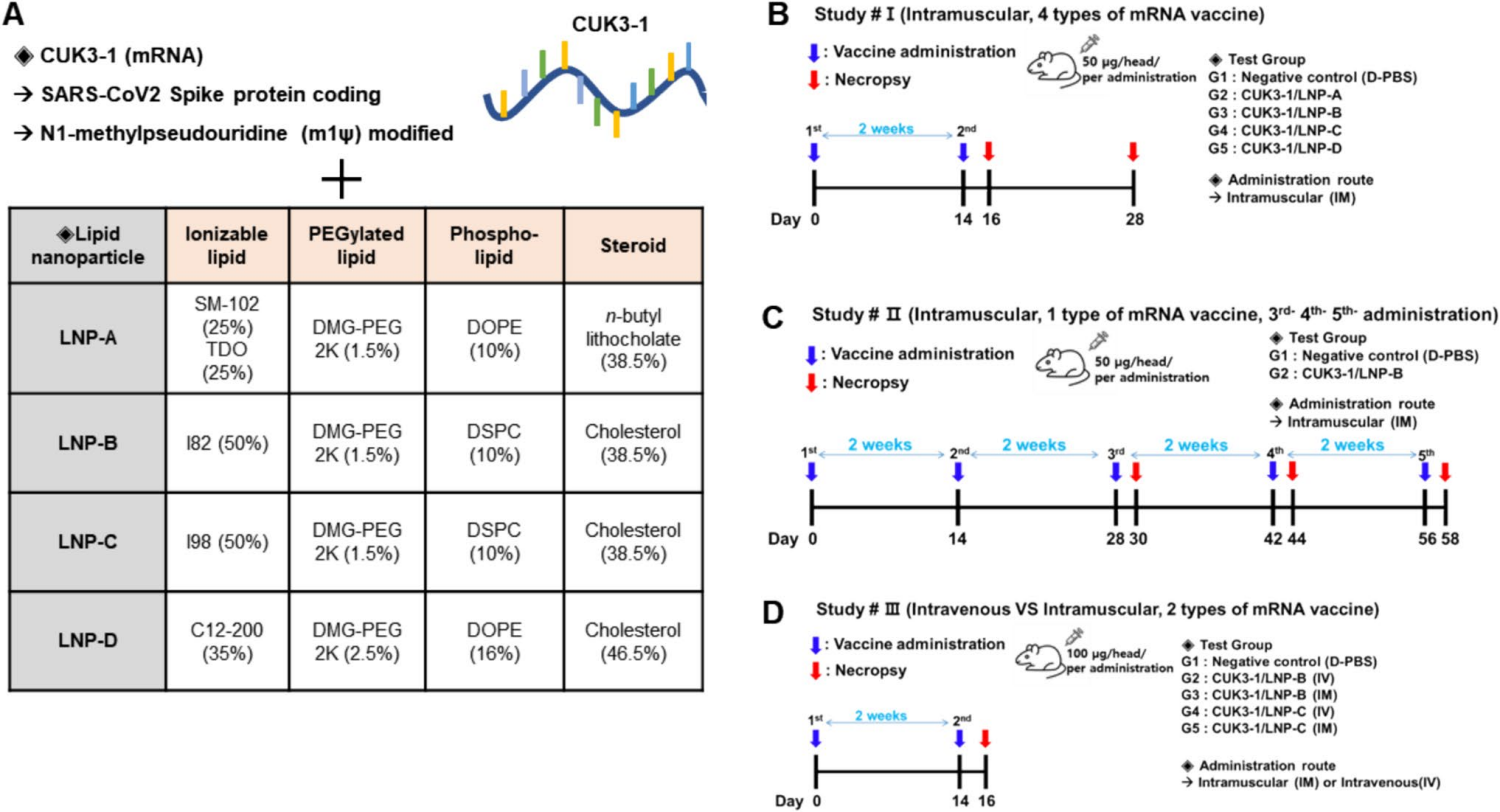
The profile of mRNA vaccine candidates and toxicity study designs. **A** Characteristics of the mRNA template (CUK3-1) and composition of four types of lipid nanoparticles. **B** Design of the animal study for acute and delayed toxicity. **C** Design of the animal study for repeated dose toxicity. **D** Design of the animal study for administration route toxicity

To examine the toxicological impacts of the mRNA vaccine, we prepared four different formulations of the SARS-CoV-2 mRNA vaccine, each with CUK3-1 combined with different LNP formulations (CUK3-1/LNP-A, CUK3-1/LNP-B, CUK3-1/LNP-C, CUK3-1/LNP-D). These vaccines were labeled A, B, C, and D in figures. For LNP-D, a liposome recognized as a standard RNA delivery vehicle was utilized (Fig. 1A). To assess toxicity, we performed a series of experiments examining physiological and immune responses, along with biochemical and histological analyses. As per the experimental design, we injected 7-week-old ICR mice with 50 or 100 μg of SARS-CoV-2 spike protein-encoding mRNA encapsulated in each LNP through prime vaccination, followed by single or multiple boosters at 2-week intervals, after which blood and tissue samples were collected (Fig. 1B–D, referred to Study #I-#III respectively). Furthermore, intravenous or intramuscular injection routes were considered in Study #III.

**Fig. 2:**
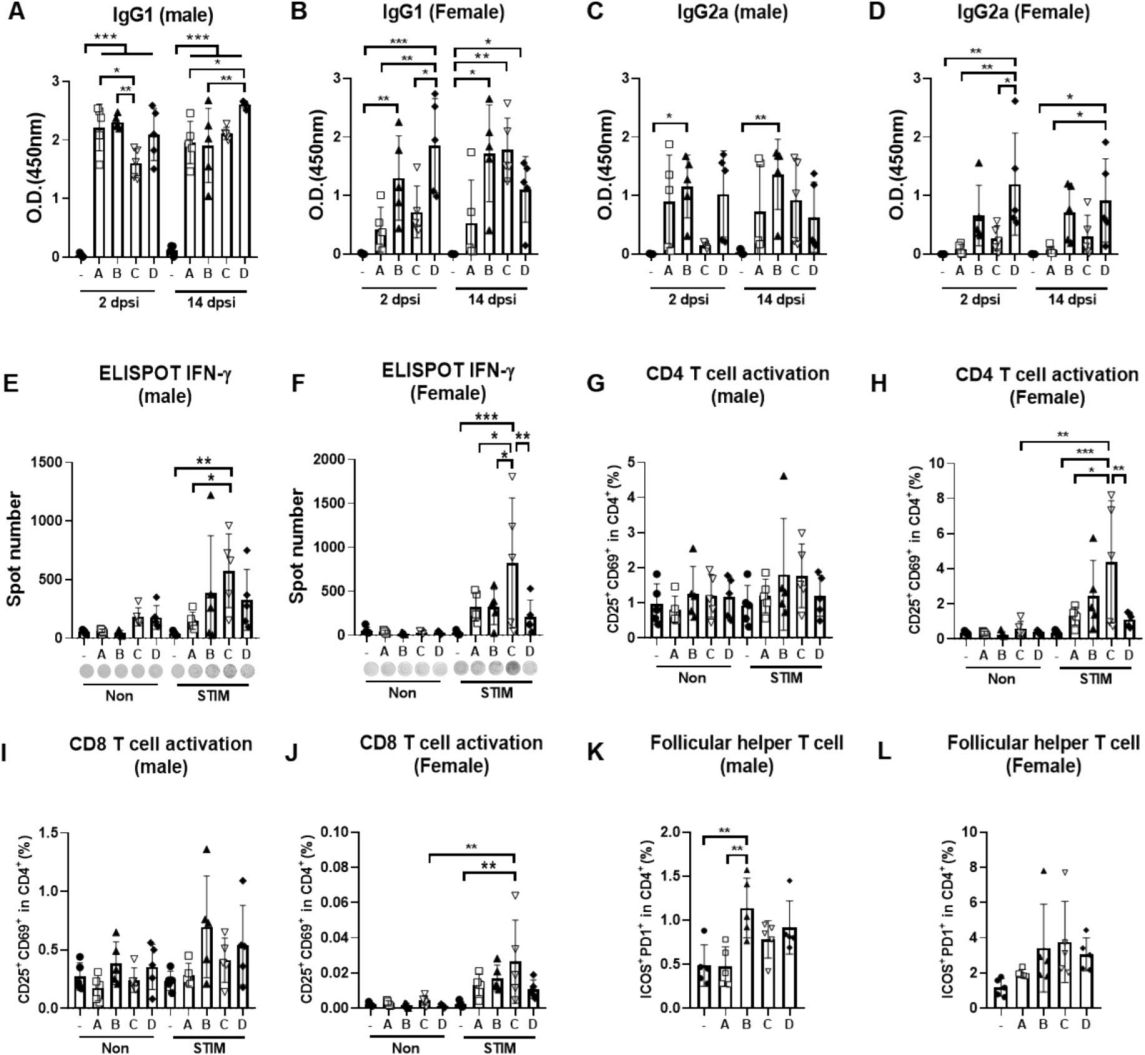
The mRNA vaccine candidates adequately elicit both SARS-CoV-2 spike protein -specific IgG and T cell activation in mice. **A**–**L** Male and female ICR mice were injected with D-PBS or specified mRNA vaccine candidates (-: D-PBS, **A** CUK3-1/LNP-A, **B** CUK3-1/LNP-B, **C** CUK3-1/LNP-C, **D** CUK3-1/LNP-D) as per Study #1. The levels of specified parameters were assessed by ELISA (**A**–**D**), ELISpot (**E**, **F**), or flow cytometry (G-L). Data were presented as mean ± SD (*n* = 5), and statistically significant differences between the negative control and each test group were analyzed using the Kruskal–Wallis test followed by the Bonferroni post hoc test (*p < 0.05, **p < 0.01, ***p < 0.001). STIM: stimulation with peptides from the SARS-CoV-2 Omicron variant spike protein (5 μg/mL)

In Study #I, we collected blood samples to evaluate the levels of SARS-CoV-2 spike protein-specific IgGs in serum at 2 dpsi and 14 dpsi, and spleen tissues were obtained to analyze T cell responses at 14 dpsi. The Th2-dependent IgG1 and Th1-dependent IgG2a antibodies were quantified using ELISA. Compared to the negative control group injected with D-PBS, all candidates increased the levels of IgG1 antibodies at 2 dpsi, which were sustained until 14 dpsi in both male and female mice (Fig. 2A, B). The CUK3-1/LNP-A or CUK3-1/LNP-B demonstrated greater increases in IgG2a levels in male mice compared to female mice. In addition, the IgG2a levels in the group receiving CUK3-1/LNP-C showed negligible or smaller changes compared to other LNP groups (Fig. 2C, D). We then evaluated the cellular immune response of the mRNA vaccine candidates in splenocytes using ELISpot and flow cytometry. All candidates enhanced IFN-γ secretion from splenocytes stimulated with spike protein peptides, relative to the D-PBS negative control (Fig. 2E, F). Notably, splenocytes from mice injected with CUK3-1/LNP-C secreted more IFN-γ than those from other groups, indicating that CUK3-1/LNP-C induced T cell activation more effectively, despite showing the lowest antibody levels among the four candidates (Fig. 2G–L). These findings suggest that the immunogenicity of mRNA vaccines varies with the type of LNP used, and our mRNA vaccine candidates function as vaccines.

### mRNA vaccine candidates induce toxicological changes at the acute phase (2 dpsi) but it was recovered at the recovery phase (14 dpsi)

Considering that BNT162b2 and mRNA-1273 have been administered in more than two doses to a person in a clinical setting, we also evaluated toxicological changes using four types of mRNA vaccine candidates in a twice-administered male and female mouse model. In addition, to ascertain the toxicity in both the acute and recovery phases, we established two endpoints: 2 dpsi and 14 dpsi (Fig. 1C).

**Fig. 3:**
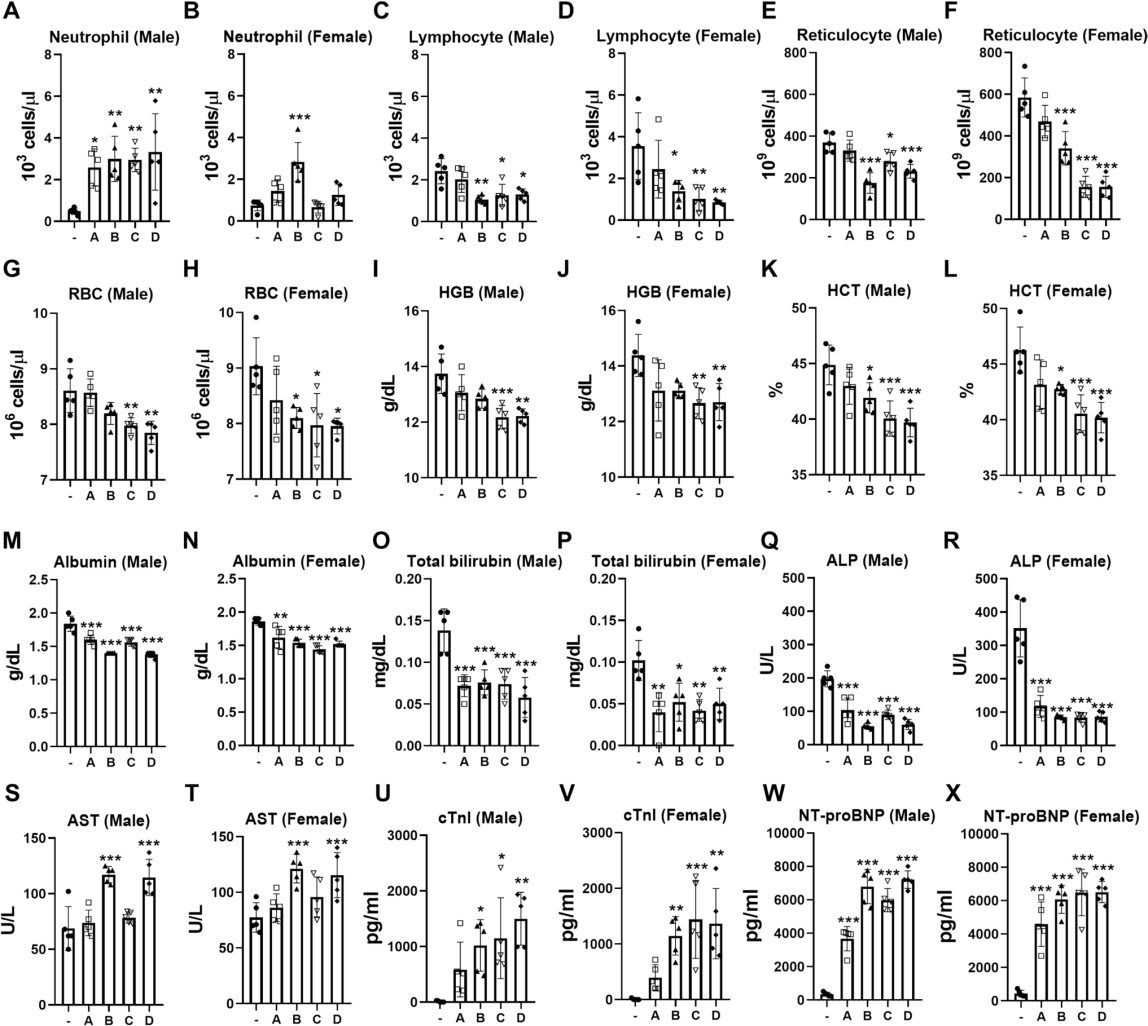
The mRNA vaccines-induced changes in hematology and blood chemistry parameters at 2-day post-second injection in Study #1. **A**–**X** Male and female ICR mice were injected with D-PBS or indicated mRNA vaccine candidates (-: D-PBS, **A** CUK3-1/LNP-A, **B** CUK3-1/LNP-B, **C** CUK3-1/LNP-C, **D** CUK3-1/LNP-D), and blood samples were collected at 2 dpsi. The levels of indicated parameters were analyzed by CBC (**A**–**L**), blood chemistry (**M**–**T**), or ELISA (**U**–**X**). Data were presented as mean ± SD (*n* = 5), and statistically significant differences between the negative control and each test group were analyzed by the Kruskal–Wallis test followed by the Bonferroni post hoc test (*p < 0.05, **p < 0.01, ***p < 0.001)

Body weight changes were monitored for 2 weeks. Although body weight slightly decreased at 1-day post each injection of mRNA vaccine candidates, compared to the negative control, the difference was not statistically significant (Supplementary data 2). CBC and blood chemistry analyses at 2 dpsi revealed several changes induced by mRNA vaccine candidates (Supplementary data 3 and 4). An increase in both the percentage and absolute count of neutrophils was observed across all test groups, with more pronounced changes in male mice than in females (Fig. 3A, B). Notably, the percentage and absolute counts of lymphocytes and reticulocytes were significantly reduced by all mRNA vaccine candidates except CUK3-1/LNP-A (Fig. 3C–F). Levels of erythrocytes, hemoglobin, and hematocrit decreased by approximately 10% in mice injected with CUK3-1/LNP-C and CUK3-1/LNP-D compared to the negative control (Fig. 3G–L). Albumin, total bilirubin, and ALP levels significantly decreased across all mRNA vaccine candidates (Fig. 3 M–R). AST levels increased following administration of CUK3-1/LNP-B and CUK3-1/LNP-D (Fig. 3S, T). In the clinical field, serum NT-proBNP and cTroponin-I are regarded as essential biomarkers for cardiac damage, because these proteins are released from damaged cardiomyocytes (Adams 3rd et al. 1993; Richards and Troughton 2004). Notably, an increase in serum NT-proBNP and cTroponin-I levels was triggered by all mRNA vaccine candidates (Fig. 3U–X and Supplementary data 4).

**Fig. 4:**
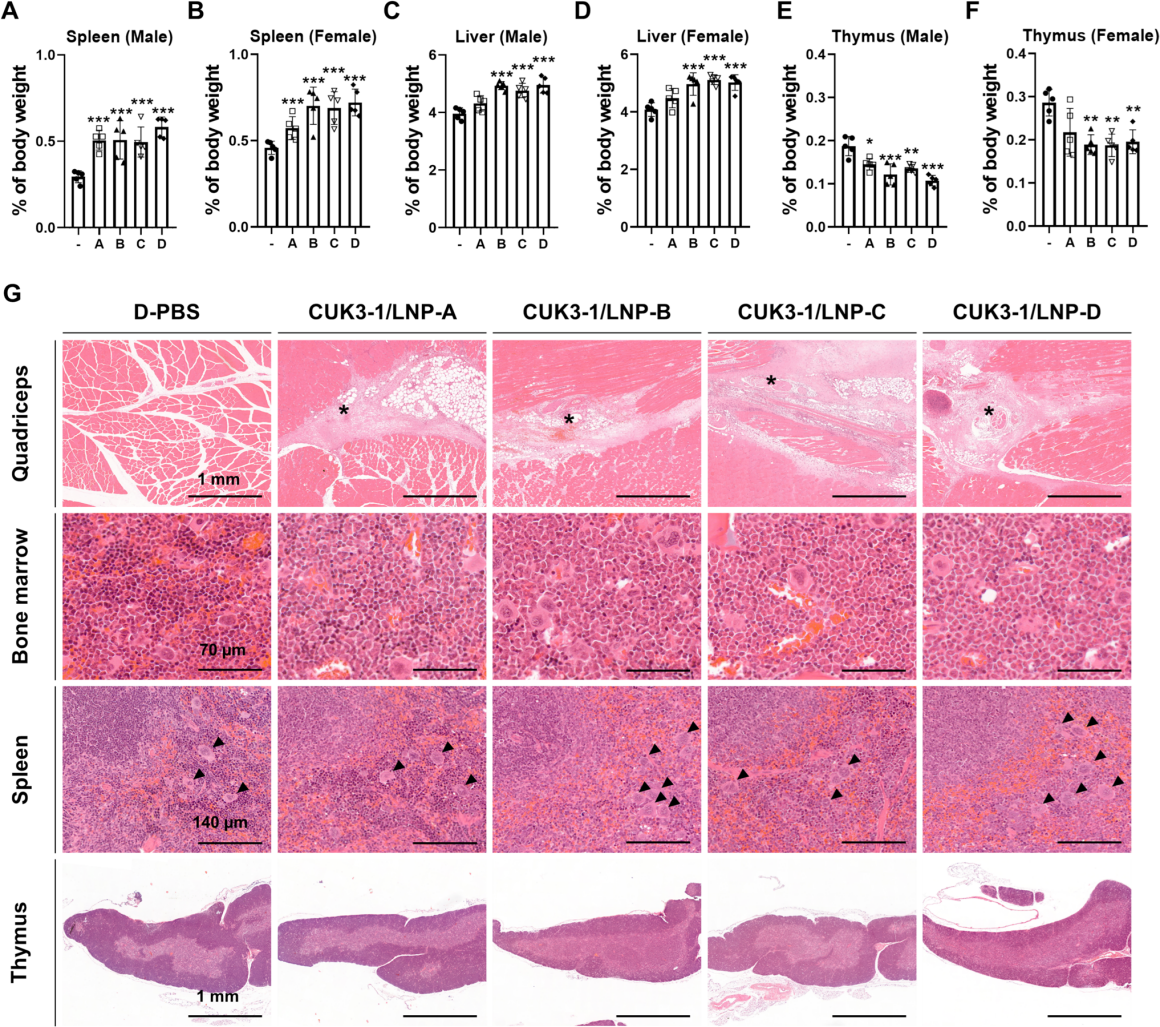
The mRNA vaccines-induced changes of relative organ weight and histopathology at 2-day post-second injection in Study #1. **A**–**G** Male and female ICR mice were injected with D-PBS or specified mRNA vaccine candidates (-: D-PBS, **A** CUK3-1/LNP-A, **B** CUK3-1/LNP-B, **C** CUK3-1/LNP-C, **D** CUK3-1/LNP-D) and necropsy was performed at 2dpsi. **A**–**F** The relative organ weights to body weight are displayed. Only the organs with statistically significant changes in relative weights were presented. The data were displayed with Mean ± SD (*n* = 5), and statistically significant differences between the negative control and each test group were analyzed using the Kruskal–Wallis test followed by the Bonferroni post hoc test (*p < 0.05, **p < 0.01, ***p < 0.001). (G) Only representative histopathological H&E sections of organs significantly affected by the mRNA vaccines are shown (Sex of mice: male; black scale bars = specified length; asterisk = Inflammation at injection sites; arrowhead = megakaryocyte)

All mRNA vaccine candidates, except CUK3-1/LNP-A, significantly increased liver and spleen weights while reducing thymus weight (Fig. 4A–F and Supplementary data 5). Histopathological analysis showed that all mRNA vaccine candidates led to an increase in lymphocyte cellularity in the spleen’s white pulp. In addition, neutrophil infiltration and increased cellularity of megakaryocytes were observed in the red pulp of the spleen, alongside a reduction in erythroid cells (Fig. 4G and Supplementary data 6). In the thymus, all groups showed cortical atrophy due to decreased lymphocyte cellularity in the cortex, most notably in the CUK3-1/LNP-B- and CUK3-1/LNP-D-injected mice, with an increase in tangible body macrophages also observed (Fig. 4G and Supplementary data 6).

All mRNA vaccine candidates induced minimal to moderate acute inflammation, characterized by infiltration of neutrophils, hemorrhage, edema, and necrosis at the injection site within the quadriceps muscle. Myofiber degeneration and/or necrosis was observed in a few mice (Fig. 4G and Supplementary data 6). Notably, we observed histopathological changes in the femur bone marrow tissue. There was a significant decrease in the number of erythroid cells in the medullary sinuses of bone marrow, with varying degrees of severity, particularly pronounced in mice injected with CUK3-1/LNP-B, CUK3-1/LNP-C, and CUK3-1/LNP-D (Fig. 4G and Supplementary data 6).

Meanwhile, most of the toxicological changes observed at 2 dpsi necropsy (Reticulocytes, lymphocytes, red blood cells, cardiac troponin-I, etc.…) had resolved by the 14 dpsi (Supplementary data 7–10). However, the increase in liver and spleen weight remained at 14 dpsi (Supplementary data 9). Furthermore, persistent chronic inflammation in the quadriceps muscle and increased cellularity of the spleen were observed at 14 dpsi in all groups, particularly in the CUK3-1/LNP-B and CUK3-1/LNP-D groups (Supplementary data 10).

These results suggest that mRNA vaccines may exhibit various potential toxicities, and the toxicological phenotype may vary depending on the LNP composition.

### Repeated dose administration of mRNA vaccine candidates reveals the unexpected toxicological changes

Considering that people have been vaccinated with two to five doses during the COVID-19 pandemic, we selected CUK3-1/LNP-B as the test substance and assessed the toxicological effects following the administration of three, four, or five repeated doses in male and female mice.

**Fig. 5:**
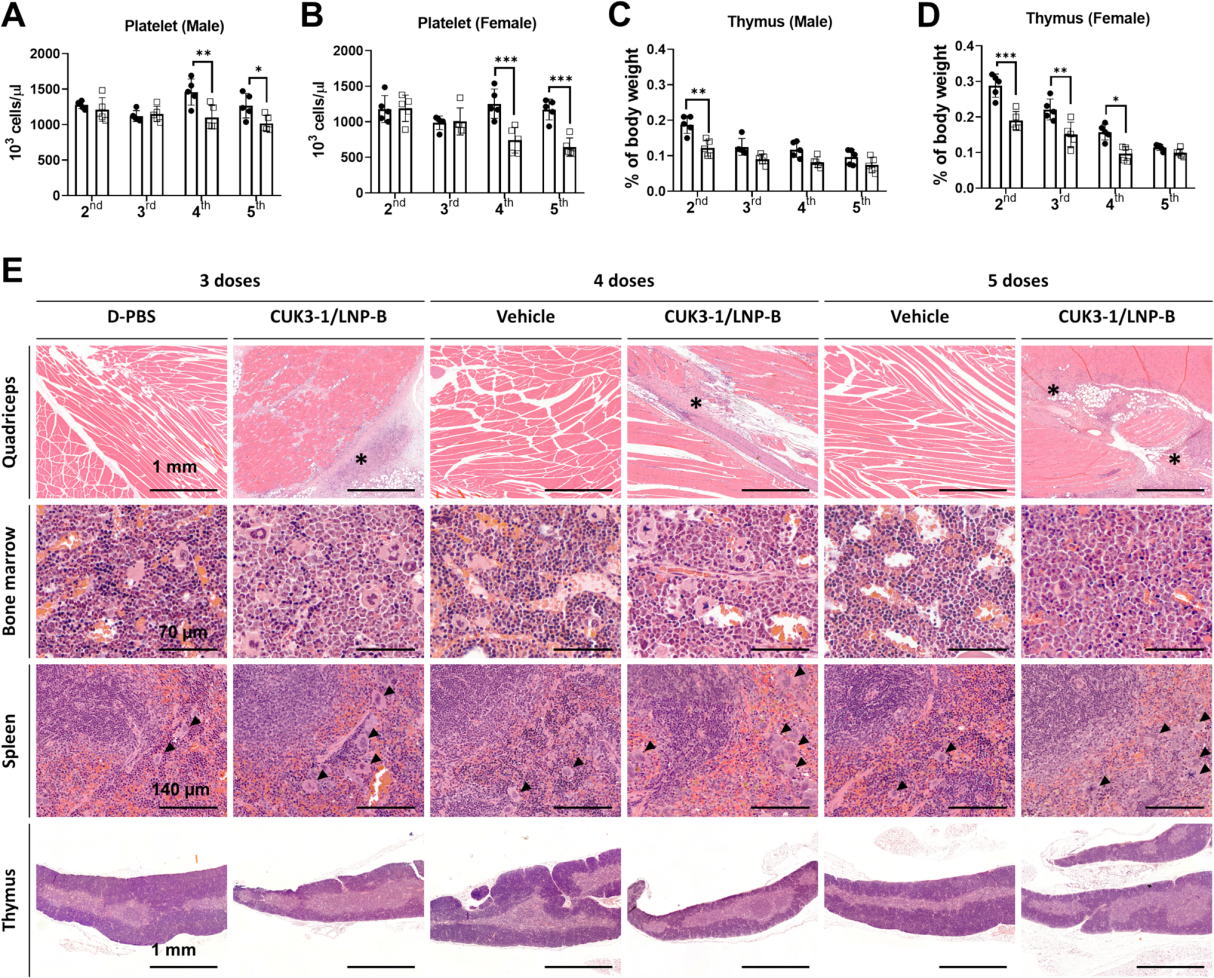
Toxicological changes following intramuscular injection of mRNA vaccine candidates with 3, 4, and 5 repeated doses at 2-day post-final injection in Study #2. **A**, **B** Circulating platelet levels in CUK3-1/LNP-B-injected male and female mice with the specified repeated doses were analyzed by CBC. **C**, **D** Thymus weight relative to body weight in CUK3-1/LNP-B-injected male and female mice with the specified doses was presented. **A**–**D** The data were presented with mean ± SD (*n* = 5) and statistically significant differences between the negative control and each test group were analyzed using the Two-way ANOVA test followed by Bonferroni’s multiple comparison post hoc test (*p < 0.05, **p < 0.01, ***p < 0.001). **E** Representative histopathological images of H&E sections of male mice (Black scale bars = indicated length; asterisk = Inflammation at injection sites; arrowhead = megakaryocyte)

The necropsy results at 2-day post-final injection from the three, four, or five doses administration groups revealed varying levels of reticulocytes, AST, ALP, cTroponin-I, liver weight, and spleen weight between the negative control and the CUK3-1/LNP-B group, comparable to those observed after two doses (Supplementary data 11A–L). Conversely, the decrease in platelet levels by CUK3-1/LNP-B, not seen in the two and three doses administration groups, was observed in the four and five doses administration groups (Fig. 5A, B). Notably, the significant difference in thymus weight between the negative control and the CUK3-1/LNP-B injected mice evident at two doses administration disappeared as the mice aged at four and five doses administration (Fig. 5C, D).

Inflammation, degeneration, and/or necrosis of myofibers at the quadriceps muscle injection site appeared to increase in severity and frequency with each administration. Furthermore, a reduction in erythroid cells was observed in the bone marrow, with the lesion’s severity increasing with repeated administrations. In the spleen, consistent with the two -dose administration groups, there was an increase in cellularity, expansion of white pulp, increased megakaryocyte and granulopoiesis cellularity, and a decrease in erythroid cell cellularity. The severity and frequency of lesions also increased with repeated administrations. With repeated administrations, minimal infiltration of monocytes became apparent in the liver parenchyma. In the thymus, cortical atrophy and increased tingible body macrophages were noted in all repeated dose groups, with more severe cortical atrophy in the four-dose than in the three-dose-injected mice. Notably, the severity of cortical atrophy was reduced in the mice injected with five doses (Fig. 5E and Supplementary data 12).

### The administration of mRNA vaccine candidates by different routes (Intravenous or intramuscular) induces different types of toxicological changes

As we confirmed that intramuscular injections of mRNA vaccine can induce several toxicological changes, we aimed to compare the toxicological phenotypes induced by intravenous (IV) versus intramuscular (IM) routes when administered in the same dosage. Generally, IV administration triggers a more severe toxic response than IM administration because it directly and rapidly delivers the test substance to the tissues and organs. Interestingly, some parameters showed greater influence from the IM route than the IV route, while others were more affected by the IV route than the IM.

**Fig. 6:**
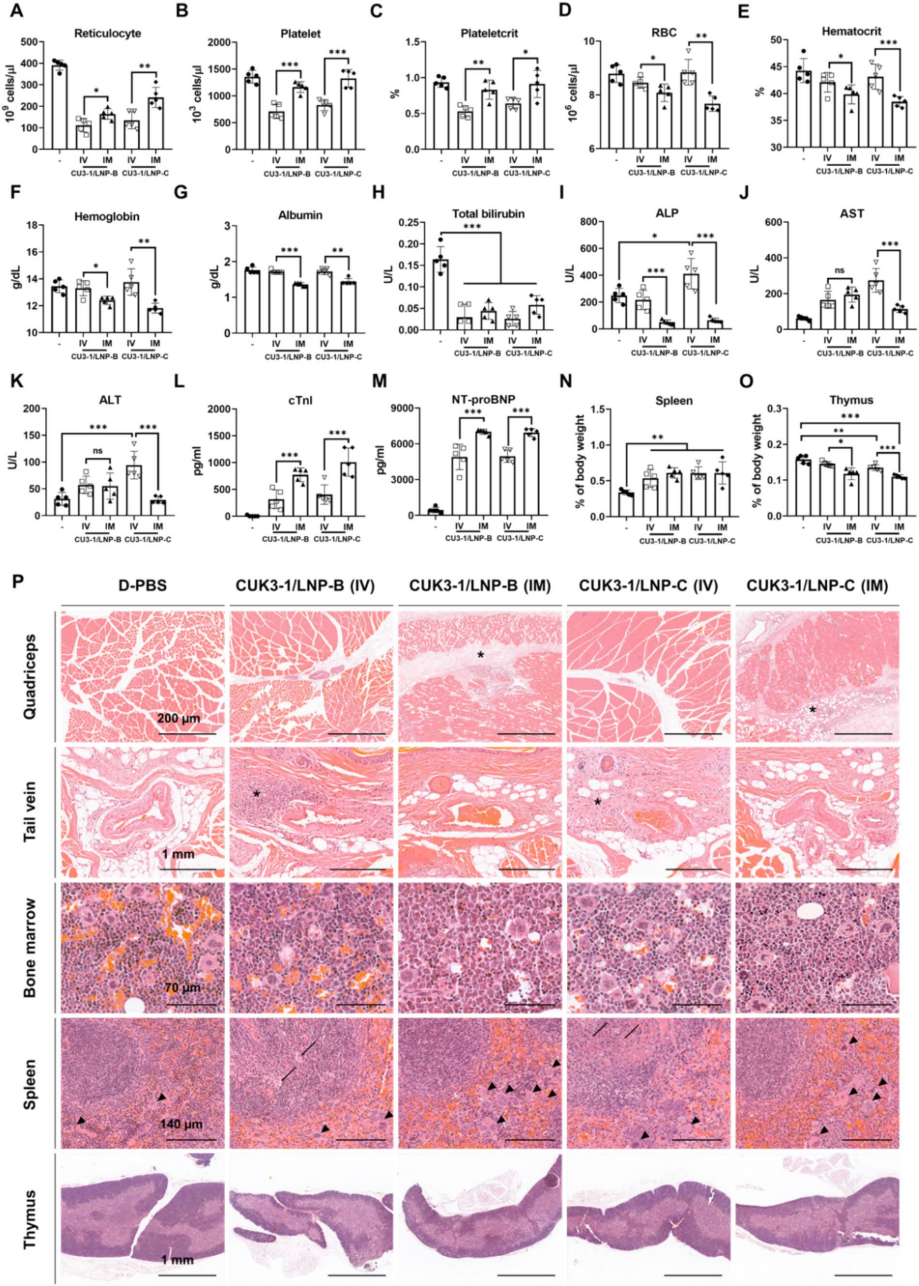
The comparative toxicological phenotype of intravenous and intramuscular injection of CUK3-1/LNP-B or CUK3-1/LNP-C at 2-day post-second injection in Study #3. **A**–**P** Male mice were injected intravenously (IV) or intramuscularly (IM) with D-PBS or mRNA vaccine candidates and samples were collected 2-day post-second injection. The levels of specified parameters in male mice were analyzed by CBC (**A**–**F**), blood chemistry (**G**–**K**), or ELISA (**L**, **M**). The indicated organ weight relative to body weight was presented (**N** and **O**). Data were presented as mean ± SD (*n* = 5), and statistically significant differences between the IV and IM groups, or between the negative control and each test group, were analyzed using the Kruskal–Wallis test followed by the Bonferroni post hoc test (*p < 0.05, **p < 0.01, ***p < 0.001). **P** Representative histopathological images of H&E sections from male mice. (Black scale bars = indicated length; asterisk = Inflammation at injection sites; arrow = apoptotic lymphocytes; arrowhead = megakaryocyte)

The reticulocyte levels were significantly decreased by mRNA vaccines in both IV and IM groups compared to the negative control group, with a more pronounced decrease observed in the IV groups (Fig. 6A). Notably, while IM administration did not affect platelet and plateletcrit levels, these levels were significantly reduced following IV administration (Fig. 6B, C). Conversely, anemia-related parameters such as RBC, hemoglobin, and hematocrit, which were reduced by IM administration, remained unaffected by IV administration (Fig. 6D–F). The albumin level only decreased in the IM administration groups, whereas total bilirubin levels decreased in both IM and IV administration groups (Fig. 6G, H). Decreases in ALP levels were solely induced by IM administration (Fig. 6I). Notably, IV administration of CUK3-1/LNP-C did not decrease ALP levels but instead caused a slight increase compared to the negative control (Fig. 6I). In CUK3-1/LNP-C injected mice, elevation of AST and ALT levels occurred only with IV administration, not IM (Fig. 6J, K). Generally, since IV administration directly targets organs, it was anticipated that mRNA vaccine-related cardiac damage would be more severe with IV administration. However, serum levels of cTnI and NT-proBNP were significantly higher in IM administration groups than in IV groups (Fig. 6L, M). In addition, the increase in spleen weight due to mRNA vaccines was similar in both IV and IM-injected mice (Fig. 6N). Conversely, thymus weight decreased more significantly with IM administration than IV (Fig. 6O).

Histopathological findings demonstrated differences in lesions between IM- and IV-injected mice. Acute inflammatory lesions were noted at the injection site in the quadriceps muscle of IM-injected mice, while perivascular inflammatory cell infiltration with minimal swelling was observed in the tail vein of IV-injected mice. Regardless of the route of administration, a decrease in erythroid cells was noted in the bone marrow across all test groups, with this effect being more pronounced in the IM groups than in the IV groups (Fig. 6P and Supplementary data 13). In the spleen, all groups exhibited increased cellularity and expansion of the white pulp, alongside a reduction in erythroid cells in the red pulp; the changes were more severe in the IV administration group. In addition, an increase of megakaryocytes and granulopoiesis in the spleen was observed solely in the IM group, while lymphocyte apoptosis in the germinal center was unique to the IV group. The thymus exhibited cortical atrophy and an increase in tangible body macrophages in both IV and IM groups. Infiltration of inflammatory cells, predominantly mononuclear, was more evident in the liver parenchyma of the IV- than the IM-injected mice (Fig. 6P and Supplementary data 13). No histopathological abnormalities were noted in the heart tissue of either group (data not shown).

## Discussion

### Need for mRNA vaccine safety assessments

Humans have long suffered from the potential toxicity of various novel agents and drugs, such as thalidomide and polyhexamethylene guanidine (PHMG) (Franks et al. 2004; Kim et al. 2016). The emergence of severe side effects from several drugs underscored the need for comprehensive and accurate safety evaluations in the drug development process. Consequently, OECD and WHO have established toxicity assessment guidelines that consider the unique characteristics of each agent. The mRNA vaccine, a critical innovation for public health against the COVID-19 pandemic, has demonstrated remarkable benefits. However, multiple side effects of mRNA vaccines have been reported, including myocarditis, thrombosis with thrombocytopenia syndrome, and Guillain–Barré syndrome (Bozkurt et al. 2021; García-Grimshaw et al. 2021; Hanson et al. 2022; Kadali et al. 2021; Sangli et al. 2021). Therefore, safety assessments for mRNA vaccines should differ from those for conventional drugs or vaccines.

### In terms of immunogenicity

Previous reports have established that administering an mRNA vaccine against COVID-19 induces IgG antibodies and IFN-γ secretion specific to the spike protein in animal models (Chaudhary et al. 2021; Zhang et al. 2020). Moreover, the effects of different administration routes and dosages on immune responses, including antibody production and T cell responses, have been investigated in conventional mRNA vaccines (Lee et al. 2023). The analysis of IgG1 and IgG2a antibodies has provided critical insights into the Th2 and Th1-dependent responses elicited by mRNA vaccine candidates against SARS-CoV-2. Notably, all candidates demonstrated a significant increase in IgG1 levels, which remained high from 2 to 14 dpsi in both male and female mice, indicative of a robust humoral response targeting the spike protein. However, there were significant disparities in IgG2a responses among the candidates; CUK3-1/LNP-A and CUK3-1/LNP-B elicited higher levels in male mice than in females, whereas CUK3-1/LNP-C showed minimal changes in IgG2a levels. In terms of cellular immune response, all candidates significantly enhanced IFN-γ secretion from splenocytes, indicating effective T cell activation compared to the negative control. Particularly noteworthy was the finding that CUK3-1/LNP-C induced higher levels of IFN-γ secretion, suggesting superior T cell activation despite lower antibody levels. These findings underscore the critical role of LNP type in mRNA vaccine formulations, affecting the immunogenicity profile. In addition, they highlight the importance of comprehensive toxicity assessments of LNPs in mRNA vaccine development to ensure both safety and efficacy.

### In terms of the hemolytic effect by LNPs

Our study revealed that mRNA vaccines induce several toxicological changes in the acute phase (2-day post-second injection). Given that the hemolytic effects of lipid nanoparticles are well-defined (Silva et al. 2014; Wang et al. 2009; Winter et al. 2016), we confirmed a mild decrease in erythrocytes, hemoglobin, and hematocrit in all mRNA vaccine-injected mice except those in the CUK3-1/LNP-A group, demonstrating that the type of LNPs dictates the hemolytic effect. The clinical cases of autoimmune hemolytic anemia following receipt of SARS-CoV2 mRNA vaccine were reported in clinical field (Brito et al. 2021; Gadi et al. 2021). It needs to be clarified whether mRNA vaccine-induced hemolysis is mediated by an autoimmune response or inflammatory damage.

### In terms of the comparative routes of administration

The commercial mRNA vaccines for SARS-CoV-2 are designed for intramuscular (IM) injection. Consequently, the pre-clinical safety assessments for mRNA-1273 and BNT162b2 were exclusively conducted through the IM route in SD rats. However, the standard safety guideline for vaccines stipulates that if toxic effects are observed with one route of administration (e.g., intramuscular), conducting further studies using an alternative route (e.g., intravenous) may elucidate the product’s full toxicity spectrum (WHO 2005). Moreover, numerous global pharmaceutical companies are developing mRNA vaccines not only for infectious diseases but also for cancer, autoimmune, and metabolic disorders, termed therapeutic mRNA vaccines (Wang et al. 2023). Significantly, to enhance efficacy, the administration route for therapeutic mRNA vaccines is considered to be intravenous (IV) (Lee et al. 2023; Li et al. 2023). In this context, we investigated the comparative toxicity of IV injections of mRNA vaccines compared to the IM route in male mice through Study #III.

Two previous studies demonstrated that IM administration of a SARS-CoV-2 mRNA vaccine candidate or BNT162b2 induces a decrease in reticulocyte levels, but not lymphocytes, in rabbits or rats (Broudic et al. 2023; Rohde et al. 2023). Notably, IM administration of our mRNA vaccine candidates, except CUK3-1/LNP-A, significantly decreased both circulating lymphocyte and reticulocyte levels. Furthermore, histopathological analysis revealed that our mRNA vaccine candidates decrease erythroid cell cellularity in bone marrow and cause cortical atrophy of the thymus. The pre-clinical studies for the approval of the COVID-19 vaccine mRNA-1273 submitted to the European Medicines Agency (EMA) reported that IM injection of mRNA vaccines induces a decrease in circulating lymphocytes and reticulocytes, as well as thymic atrophy (Moderna 2021). Interestingly, IV administration of our mRNA vaccine candidates more significantly reduced the levels of circulating lymphocytes and reticulocytes than IM administration, but decreases in erythroid cell cellularity in bone marrow and cortical atrophy of the thymus were more pronounced in IM-injected mice than in IV-injected mice. The decrease of anemia-related parameters was only observed in IM-injected mice, coinciding with the phenotype of decreased pattern of erythroid cells in the bone marrow. Conversely, the decrease in circulating platelet levels was confirmed in IV- but not in IM-injected mice. These results suggest that the decrease in circulating blood cells, except erythrocytes, may be mediated by the direct distribution of the mRNA vaccine in the bloodstream, whereas pathological changes in bone marrow and thymus might be mediated by certain mechanisms related to the vaccine's specific distribution into the tissue. Meanwhile, the discordant pattern of circulating erythrocyte or reticulocyte-lymphocyte decrease between IV and IM administration raises suspicion that the decrease of reticulocyte-lymphocyte may not be mediated by lipid nanoparticles. Although we have not demonstrated whether the mRNA vaccine-induced decrease in circulating lymphocytes and reticulocytes is caused by direct damage to the original source (thymus and bone marrow) or by a direct cytotoxic effect on circulating blood cells, these toxicological changes should not be overlooked and require comprehensive investigation in follow-up studies. Furthermore, it should be noted that the administration route of mRNA vaccine can determine the toxic phenotype.

### In terms of the changes of ALP and bilirubin levels

In all IM-injected mice, mRNA vaccines significantly reduced serum ALP and total bilirubin levels, both of which are closely associated with hepatic function (Ozer et al. 2008). While elevated levels of ALP and bilirubin typically indicate liver or gallbladder dysfunction, decreasing levels are seldom considered significant in clinical practice (Sharma et al. 2014). Notably, in IV-injected mice, only total bilirubin levels were reduced, suggesting different mechanisms for the reduction of ALP and bilirubin. Despite not identifying the mechanisms by which mRNA vaccines reduced ALP and bilirubin levels, further investigation into the underlying processes is required.

### In terms of the repeated doses administration

In the clinical field, individuals received two to five doses of the SARS-CoV-2 mRNA vaccine. Generally, chemical or drug-induced toxicity accumulates with repeated administration. Notably, several studies have shown that repeated administration of mRNA vaccines increases the incidence of side effects in the clinical field (Larson et al. 2021; Merchant 2021; Riad et al. 2021). However, no studies have yet confirmed differences in toxicological phenotypes by the mRNA vaccine dependent on the number of administrations. Study #II revealed that intramuscular administration of CUK3-1/LNP-B with four and five doses reduces circulating platelet levels, but not with two and three doses. mRNA vaccine-induced decrease in circulating platelet levels was also reported in the BNT162b2 approval document and one research article (FDA 2022a, b; Rohde et al. 2023). Several studies have identified vaccine-induced thrombosis with thrombocytopenia (VITT) as a rare side effect of mRNA vaccines (Parums 2021; Sangli et al. 2021). Circulating platelets (= thrombocytes) are formed and released by megakaryocytes, which differentiate from bone marrow progenitor cells (Patel et al. 2005). Thus, the primary pathogenesis of thrombocytopenia is considered direct damage to circulating platelets or suppression of bone marrow progenitor cells (Krishnegowda and Rajashekaraiah 2015). Furthermore, the results of Study #III showed that IV administration of mRNA vaccines with two doses—not IM—significantly induces a decrease in circulating platelet levels. Meanwhile, bone marrow suppression was more pronounced in IM groups than in IV groups. These findings suggest that the reduction in circulating platelets by mRNA vaccines might be mediated by direct damage to the circulating cells rather than to the bone marrow. Intriguingly, it has been reliably suggested that the pathophysiology of COVID-19 VITT is mediated by non-specific antibody responses, particularly PF4-reactive antibodies (Lai et al. 2021). Some previous studies suggested that COVID-19 VITT is mediated by the antigen of COVID-19, spike protein (Greinacher et al. 2021; Kowarz et al. 2022). However, our prior research confirmed that intramuscular administration of a luciferase-coding wild-type mRNA (Methylpseudouridine unmodified) formulated with lipid nanoparticles, which does not produce any SARS-CoV2 antigen, also significantly reduces circulating platelet levels in mice (Data not shown). In fact, vaccine-induced thrombocytopenia is not only caused by the COVID-19 vaccine but also by the vaccines targeting other pathogens (Grimaldi-Bensouda et al. 2012; Hamiel et al. 2016; Perricone et al. 2014). Of note, regardless of the pathogen type, the vaccines have a common biomaterial, nucleic acid (live or attenuated vaccine → Viral genome; Adenovirus vector vaccine → Adenovirus genome; DNA vaccine → DNA template; mRNA vaccine → mRNA template). Furthermore, when nucleic acid stimulates the pattern recognition receptors in platelets, the platelet is activated and consumed, and thrombosis is promoted (Hally et al. 2017; Koupenova et al. 2014; Panigrahi et al. 2013). Although the specific mechanism of VITT is not fully identified, it is suspected that the antigen of vaccine is not involved in the VITT etiology. These pieces of evidence strongly indicate that the mechanism underlying the SARS-CoV-2 mRNA vaccine-induced decrease in platelet levels may not be mediated by the SARS-CoV-2 antigen or antibodies, necessitating further clarification through follow-up studies.

### In terms of thymic atrophy

Thymic atrophy was also reported in the BNT162b2 approval document demonstrating that BNT162b1 or BNT162b2-injected rats showed decreased thymus weight compared to control mice with a 14–28% (FDA 2022a, b). We confirmed that intramuscular administration of our mRNA vaccine candidates induces thymic atrophy and a decrease in thymus weight in mice receiving two doses. Interestingly, this decrease in thymus weight was less apparent in mice injected with three to five doses (male) and five doses (female), compared to those receiving D-PBS. However, cortical atrophy persisted and was more pronounced in mice receiving four and five doses compared to those with two doses. Notably, as the number of administrations increased, so did the age of the mice at the time of necropsy, since the first administration of the mRNA vaccine was given at 7 weeks old across all repeated dose groups. The thymus, a critical primary lymphoid organ for establishing lymphocyte-mediated immunity in early life, gradually degenerates with aging (Bodey et al. 1997). Therefore, it is reliably suspected that the absence of observed thymus weight loss in the multi-dose mRNA vaccine-injected mice, compared to same-age D-PBS-injected mice, was due to concurrent thymus degeneration with aging in both D-PBS- and mRNA vaccine-injected mice. Indeed, the pattern of thymus weight loss due to aging in D-PBS-injected mice was comparable to reference data in mice (Liu et al. 2012). Our findings suggest that mRNA vaccines may significantly affect the thymus at younger ages in terms of toxicity. Previous studies also show that the thymus is more vulnerable to toxic agents at younger rather than older ages (Bach 1977; Dortant et al. 2001; Kuper et al. 1992). Importantly, maintaining thymic function at young ages is crucial for establishing the memory function of T lymphocytes (Linton and Dorshkind 2004). Thus, altered thymic activity at younger ages could lead to a decline in immune function in both young adults and older individuals (Sauce and Appay 2011). These pieces of evidence strongly suggest that the potential juvenile toxicity of mRNA vaccines in terms of immune function needs comprehensive identification through follow-up studies.

### In terms of elevated cardiac damage markers

Myocarditis and pericarditis are the most notable side effects of the SARS-CoV-2 mRNA vaccine in the clinical field (Bozkurt et al. 2021; Kim et al. 2021; Larson et al. 2021; Switzer and Loeb 2022). Unfortunately, in the pre-clinical safety assessment guidelines, mandatory biomarkers for predicting myocarditis and pericarditis have not been established. Therefore, we evaluated the potential for mRNA vaccine-induced myocarditis in mice by measuring cardiac Troponin-I (cTnI) and NT-proBNP, which are essential biomarkers for diagnosing myocarditis and pericarditis in the clinical field. Notably, IM administration of our mRNA vaccine candidates significantly increased the release of cTnI and NT-proBNP, yet histopathological changes in cardiac tissue were not observed in H&E stain and F4. One previous experimental study demonstrated that IV administration of the mRNA vaccine (BNT162b2) induces the myocarditis phenotype more significantly than IM administration in Balb/c mice (Li et al. 2022). However, we could not find any mRNA vaccine-induced histopathological changes in the hearts of either IM-injected or IV-injected mice. There has been debate regarding the release of cTnI in reversible cardiac damage. While one study showed that reversible ischemia does not induce the release of cTnI (Carlson et al. 2002), other studies have revealed that even reversible cardiac ischemia secretes cTnI into the blood (Feng et al. 1998; Wu and Ford 1999). In addition, in cases of non-fatal damage, a transient release of cTnI can occur without significant damage to cardiomyocytes (Neumayr et al. 2001; Rifai et al. 1999). This evidence suggests that mRNA vaccines can damage cardiomyocytes and induce the release of cTnI and NT-proBNP, though histopathological changes in cardiac muscle may vary based on susceptible doses, host species, type of mRNA vaccine, administration route, and other experimental conditions. Interestingly, our results showed that the increase in serum cTnI and NT-proBNP levels was significantly higher in IM-injected mice compared to IV-injected mice. This finding is inconsistent with the previous experimental study, which found that IV administration of BNT162b2 induces myocarditis more significantly than IM administration (Li et al. 2022). Importantly, when LNPs are injected into the bloodstream, they interact with Apolipoprotein E (ApoE), which is highly conserved in hepatocytes, leading to rapid absorption and degradation of LNPs in the liver (Pardi et al. 2015; Yan et al. 2005). Consequently, mRNA-LNP agents degrade more rapidly when injected intravenously compared to intramuscularly (Pardi et al. 2015). Exceptionally, the ApoE-mediated absorption and degradation of LNPs can vary based on the LNP composition, and ApoE-independent pathways have also been identified (Gregersen et al. 2024; Paunovska et al. 2022; Sebastiani et al. 2021). LNPs are delivered to various tissues from the injection site via not only blood vessels but also lymphatic vessels (McCright et al. 2022; Rip et al. 2005). Lymphatic vessels, well-conserved in skeletal muscle and heart, play a crucial role in tissue homeostasis (Henri et al. 2016; Kivelä et al. 2007; Klotz et al. 2015). Therefore, we hypothesize that when the mRNA vaccines, composed of LNPs susceptible to ApoE-mediated degradation administered via the IM route, the mRNA vaccines might remain in the body than the IV route and affect heart tissue more via lymphatic vessels than blood vessels. This hypothesis needs clarification through follow-up studies. For the specializing study in terms of mRNA vaccine-induced myocarditis, noninvasive cardiac function tests (e.g., echocardiography) and in vitro cardiomyocyte experiments would be helpful to clarify the underlying toxicity mechanism.

### In terms of sex-specific differences of toxic phenotypes

Numerous reports suggest that SARS-CoV-2 mRNA vaccines may induce disorders in the female reproductive cycle (Gibson et al. 2022; Hajjo et al. 2023; Seyfi-Ghale-Jogh et al. 2023). However, our findings show no toxicological alterations in the reproductive systems (ovary, uterus, vagina) of mice, nor any significant differences in mRNA vaccine-induced toxicity between male and female mice. It is important to note that our study did not include specific analyses on the reproductive cycle or hormonal changes. To determine the potential reproductive toxicity of mRNA vaccines, targeted follow-up studies such as developmental and reproductive toxicology (DART) tests are necessary. Immunogenicity is closely related to the side effects (Reactogenicity) of the vaccines, and females generally exhibit higher immunogenicity than males (Klein et al. 2015; Fischinger et al. 2019). That is why sex-specific differences in vaccine-induced toxicity would be comprehensively considered in toxicity studies. Therefore, identifying the sex-specific immunogenicity and reactogenicity in terms of hormonal or genetic factors would be helpful to secure the safety and efficacy of mRNA vaccines, and this could lead to personalized vaccination strategies where dosing or formulation varies by sex.

### In terms of the recovery of toxic phenotype for 14-day post-secondary injection

All toxicological alterations caused by two administrations of mRNA vaccines at 2-day post-injection were reversed by 14-day post-secondary injection, except for pathological changes at the injection site. Despite the local toxicity being triggered by administering the highest concentration and dose of mRNA vaccine candidates relative to mouse body surface area, the significance of mRNA vaccine-induced local toxicity must not be underestimated.

### Supplement point

In this study, we comprehensively identified the potential toxicity of mRNA vaccines via pre-clinical toxicity tests. However, there are also several limitations. First, we did not conduct the dose–response toxicity test, so we could not demonstrate the dose dependency of the toxic changes. Second, we did not identify the detailed toxicological conditions, such as no-observed-adverse-effect level (NOAEL), 50% lethal dose (LD 50%), maximum tolerated dose (MTD) and the effect of dosing intervals. Lastly, we just presumed, not demonstrated, the mechanism of the mRNA vaccine-induced several toxic phenotypes. These limitations are assignments that we must identify via follow-up studies.

### Summary and future direction

In summary, our investigation into the toxicological effects of four SARS-CoV-2 mRNA vaccine candidates revealed various toxicological changes. We also discovered that different administration routes lead to distinct toxicological phenotypes and that repeated doses can accumulate toxicity. Although the specific mechanisms behind each toxic change were not delineated, our study provides valuable insights that may assist in the development of new mRNA vaccines. We will further investigate the limitations of this study and toxicity mechanism regarding each phenotype (e.g., decrease in circulating blood cells, elevated cardiac damage markers, bone marrow suppression, thymic atrophy) via follow-up studies.

## Supplementary Information

Below is the link to the electronic supplementary material.

Supplementary file1 (DOCX 190 KB)

## Data Availability

The datasets used and analyzed during the current study are available from the corresponding author upon reasonable request.

## Abbreviations

MCV: Mean corpuscular volume
MCH: Mean corpuscular hemoglobin
MCHC: Mean corpuscular hemoglobin concentration
BUN: Blood urea nitrogen
ALP: Alkaline phosphatase
AST: Aspartate transaminase
ALT: Alanine transaminase
CPK: Creatine phosphokinase
HDL: High-density lipoprotein
LDL: Low-density lipoprotein
cTn1: Cardiac troponin-I
NT-proBNP: N-terminal pro-brain natriuretic peptide
LNP: Lipid nanoparticle
FACS: Flow cytometry
ELISA: Enzyme-linked immunosorbent assay
ELISpot: Enzyme-linked immunospot
CBC: Complete blood count
RBC: Red blood cell
EMA: European Medicines Agency
FDA: Food and Drug Administration
