# Supplementary material for: Impact of administration routes and dose frequency on the toxicology of SARS-CoV-2 mRNA vaccines in mice model
Source: Arch Toxicol. 2024 Dec 10;99(2):755–73. doi: 10.1007/s00204-024-03912-1 (PMC11775000; doi:10.1007/s00204-024-03912-1)
Supplement: Supplementary file 1 — Supplementary file1 (DOCX 190 KB) [file 204_2024_3912_MOESM1_ESM.docx]

**Journal name: Archives in Toxicology**

**Supplementary Information**

**Article Title: Pre-clinical safety assessment in acute and recovery phase for 4 types of SARS-CoV-2 mRNA vaccine candidates in ICR mice: regarding efficacy, repeated doses and administration route.**

Jae-Hun Ahn^1#^, Jisun Lee^4#^, Gahyun Roh^4,5#^, Na-Young Lee^1,3^, Hee-Jin Bae^1^,Euna Kwon^1^, Hyo-Jung Park^4,5^, Soyeon Yoo^6^, Sung Pil Kwon^6^, Eun-Kyoung Bang^6^, Gyochang Keum6 Jae-Hwan Nam^4,5*^ and Byeong-Cheol Kang^1,2,3*^

^1^Department of Experiment Animal Research, Biomedical Research Institute, Seoul National University Hospital, Seoul, Republic of Korea

^2^Graduate School of Translational Medicine, Seoul National University College of Medicine, Seoul, Republic of Korea

^3^Department of Veterinary Pathology and Research Institute of Veterinary Science, College of Veterinary Medicine, Seoul National University, Seoul, Korea

^4^Department of Medical and Biological Sciences, The Catholic University of Korea, Gyeonggi-do, Bucheon, Republic of Korea

^5^BK Four Department of Biotechnology, The Catholic University of Korea, Gyeonggi-do, Bucheon, Republic of Korea

^6^Center for Brain Technology, Brain Science Institute, Korea Institute of Science and Technology, Seoul, Republic of Korea

^#^these authors equally contributed to this work as a first author.

**^*^**Correspondence to:

Byeong-Cheol Kang, DVM and Ph.D.

Laboratory Animal Medicine, College of Medicine, Seoul National University, 103, Daehak-ro Jongno-gu, Seoul, Republic of Korea; Tel.: +82-2-2072-0841; E-mail address: bckang@snu.ac.kr

Jae-Hwan Nam, Ph.D.

Department of Medical and Biological Sciences, The Catholic University of Korea, 43 Jibong-ro, Bucheon, Gyeonggi-do 14662, Republic of Korea; Tel.: +82-2-2164-4852; Fax: +82-2-2164-4865; E-mail: jhnam@catholic.ac.kr

| **(A) The patent information for LNP synthesis** | | | |
| --- | --- | --- | --- |
| **Contents** | **KR** | **PCT** | **US** |
| *n*-butyl lithocholate and TDO | No. 10-2021-0153940 | No. PCT/KR2022/02372 | No. 17/676822 |
| I82 and I98 | No. 10-2023-0136909 | No. PCT/KR2023/016630 | No. 18/536271 |

| **(B) The structural and physicochemical properties of mRNA vaccine candidates** | | | | |
| --- | --- | --- | --- | --- |
| **Contents** | **Z-average (nm)** | **Polydispersity index** | **Zeta potential (mV)** | **Encapsulation efficiency (%)** |
| CUK3-1/LNP-A | 97.4 ± 1.4 | 0.096 | 11.7 ± 2.0 | 87.9 |
| CUK3-1/LNP-B | 132.2 ± 1.7 | 0.080 | -3.8 ± 0.7 | 93.3 |
| CUK3-1/LNP-C | 137.1 ± 1.6 | 0.076 | -13.1 ± 1.0 | 87.7 |
| CUK3-1/LNP-D | 125.3 ± 0.9 | 0.12 | 3.5 ± 3.0 | 79.2 |

**Supplementary data 1. The patent information for LNPs and the physicochemical properties of CUK3-1/LNPs.**

**(A)** The patent information regarding LNP synthesis in the Republic of Korea (KR), Patent Cooperation Treaty (PCT), and United States (US) is detailed. **(B)** The structural and physicochemical properties of each CUK3-1/LNPs were determined by Zetasizer Ultra and RiboGreen assay. The data were presented with Mean ± SD (n=3).

| **<Study #1> Group Summary of Body Weight (Unit : g)^1^** | | | | | | | | | | | | | | | | | | | | | | | | | | | | | | | | | | | | | | | | |
| --- | --- | --- | --- | --- | --- | --- | --- | --- | --- | --- | --- | --- | --- | --- | --- | --- | --- | --- | --- | --- | --- | --- | --- | --- | --- | --- | --- | --- | --- | --- | --- | --- | --- | --- | --- | --- | --- | --- | --- | --- |
| **Sex** | **Male** | | | | | | | | | | | | | | | | | | | | **Female** | | | | | | | | | | | | | | | | | | | |
| **Test Item** | **D-PBS** | | | | **CUK3-1/LNP-A** | | | | **CUK3-1/LNP-B** | | | | **CUK3-1/LNP-C** | | | | **CUK3-1/LNP-D** | | | | **D-PBS** | | | | **CUK3-1/LNP-A** | | | | **CUK3-1/LNP-B** | | | | **CUK3-1/LNP-C** | | | | **CUK3-1/LNP-D** | | | |
| **mRNA Dosage**  **(μg/head)** | **0** | | | | **50** | | | | **50** | | | | **50** | | | | **50** | | | | **0** | | | | **50** | | | | **50** | | | | **50** | | | | **50** | | | |
| **Day Post Injection** | **MEAN** |  | **S.D.** | **N** | **MEAN** |  | **S.D.** | **N** | **MEAN** |  | **S.D.** | **N** | **MEAN** |  | **S.D.** | **N** | **MEAN** |  | **S.D.** | **N** | **MEAN** |  | **S.D.** | **N** | **MEAN** |  | **S.D.** | **N** | **MEAN** |  | **S.D.** | **N** | **MEAN** |  | **S.D.** | **N** | **MEAN** |  | **S.D.** | **N** |
| **0^1st^** | 35.10 | ± | 1.46 | 5 | 34.80 | ± | 1.16 | 5 | 34.63 | ± | 1.15 | 5 | 34.48 | ± | 1.05 | 5 | 34.97 | ± | 1.35 | 5 | 26.23 | ± | 0.94 | 5 | 27.02 | ± | 0.76 | 5 | 26.89 | ± | 0.50 | 5 | 26.53 | ± | 0.79 | 5 | 26.61 | ± | 0.79 | 5 |
| **1** | 35.12 | ± | 1.24 | 5 | 34.31 | ± | 1.10 | 5 | 33.61 | ± | 1.39 | 5 | 33.45 | ± | 1.35 | 5 | 32.96 | ± | 0.71 | 5 | 25.83 | ± | 0.87 | 5 | 26.29 | ± | 1.08 | 5 | 25.48 | ± | 0.57 | 5 | 25.02 | ± | 0.81 | 5 | 25.10 | ± | 0.77 | 5 |
| **2** | 35.70 | ± | 1.25 | 5 | 35.19 | ± | 1.23 | 5 | 34.32 | ± | 1.35 | 5 | 34.11 | ± | 1.02 | 5 | 33.87 | ± | 1.03 | 5 | 26.22 | ± | 0.78 | 5 | 26.48 | ± | 1.20 | 5 | 26.12 | ± | 0.43 | 5 | 25.34 | ± | 0.72 | 5 | 25.61 | ± | 0.46 | 5 |
| **3** | 35.70 | ± | 1.46 | 5 | 35.33 | ± | 1.88 | 5 | 34.91 | ± | 1.22 | 5 | 34.78 | ± | 1.11 | 5 | 34.41 | ± | 1.62 | 5 | 26.59 | ± | 0.84 | 5 | 27.05 | ± | 1.27 | 5 | 27.09 | ± | 0.70 | 5 | 26.46 | ± | 0.71 | 5 | 26.40 | ± | 0.38 | 5 |
| **7** | 37.12 | ± | 1.63 | 5 | 36.66 | ± | 1.67 | 5 | 36.17 | ± | 1.21 | 5 | 36.28 | ± | 0.76 | 5 | 35.35 | ± | 2.11 | 5 | 27.33 | ± | 1.02 | 5 | 27.98 | ± | 1.34 | 5 | 27.96 | ± | 0.56 | 5 | 27.36 | ± | 1.09 | 5 | 27.29 | ± | 0.95 | 5 |
| **14^2nd^** | 39.29 | ± | 2.23 | 5 | 37.91 | ± | 1.79 | 5 | 38.20 | ± | 1.09 | 5 | 38.28 | ± | 1.30 | 5 | 38.19 | ± | 2.53 | 5 | 29.29 | ± | 1.41 | 5 | 29.59 | ± | 1.06 | 5 | 29.85 | ± | 1.74 | 5 | 28.81 | ± | 1.07 | 5 | 28.45 | ± | 0.57 | 5 |
| **15** | 39.17 | ± | 2.29 | 5 | 37.83 | ± | 1.45 | 5 | 36.84 | ± | 1.89 | 5 | 37.29 | ± | 1.33 | 5 | 36.61 | ± | 2.29 | 5 | 28.62 | ± | 1.39 | 5 | 28.63 | ± | 1.02 | 5 | 28.75 | ± | 1.49 | 5 | 27.54 | ± | 1.36 | 5 | 27.47 | ± | 0.59 | 5 |
| **16^2^** | 35.15 | ± | 1.94 | 5 | 34.43 | ± | 1.99 | 5 | 34.48 | ± | 2.20 | 5 | 34.07 | ± | 1.37 | 5 | 34.19 | ± | 2.39 | 5 | 25.67 | ± | 1.73 | 5 | 26.16 | ± | 1.18 | 5 | 26.62 | ± | 1.59 | 5 | 25.25 | ± | 1.68 | 5 | 25.22 | ± | 0.79 | 5 |
| **S.D., Standard Deviation** | | | | | | | | | | | | | | | | | | | | | | | | | | | | | | | | | | | | | | | | |
| **N, Number of Animals** | | | | | | | | | | | | | | | | | | | | | | | | | | | | | | | | | | | | | | | | |
| **^1^, No statistical significance was observed** | | | | | | | | | | | | | | | | | | | | | | | | | | | | | | | | | | | | | | | | |
| **^2^, The day of necropsy (Fasting for 15 hours)** | | | | | | | | | | | | | | | | | | | | | | | | | | | | | | | | | | | | | | | | |
| **^1st^, First administration of mRNA vaccine** | | | | | | | | | | | | | | | | | | | | | | | | | | | | | | | | | | | | | | | | |
| **^2nd^, Second administration of mRNA vaccine** | | | | | | | | | | | | | | | | | | | | | | | | | | | | | | | | | | | | | | | | |

**Supplementary data 2. Group summary of body weight changes in study #1 (2 day post second injection)** Body weight measurements of mice were taken on the specified day post injection. The data were presented with Mean ± SD (n=5), and the statistically significant differences between the negative control and each test group were analyzed using Two-way ANOVA followed by Bonferroni’s multiple post hoc test. Statistically significant differences were not observed between D-PBS- and mRNA vaccine-injected mice.

| **<Study #1> Group Summary of Hematology (2 day post second injection)** | | | | | | | | | | | | | | | | | | | | | | | | | | | | | | | | | | | | | | | | |
| --- | --- | --- | --- | --- | --- | --- | --- | --- | --- | --- | --- | --- | --- | --- | --- | --- | --- | --- | --- | --- | --- | --- | --- | --- | --- | --- | --- | --- | --- | --- | --- | --- | --- | --- | --- | --- | --- | --- | --- | --- |
| **Sex** | **Male** | | | | | | | | | | | | | | | | | | | | **Female** | | | | | | | | | | | | | | | | | | | |
| **Test Item** | **D-PBS** | | | | **CUK3-1/LNP-A** | | | | **CUK3-1/LNP-B** | | | | **CUK3-1/LNP-C** | | | | **CUK3-1/LNP-D** | | | | **D-PBS** | | | | **CUK3-1/LNP-A** | | | | **CUK3-1/LNP-B** | | | | **CUK3-1/LNP-C** | | | | **CUK3-1/LNP-D** | | | |
| **mRNA Dosage (μg/head)** | **0** | | | | **50** | | | | **50** | | | | **50** | | | | **50** | | | | **0** | | | | **50** | | | | **50** | | | | **50** | | | | **50** | | | |
| **Parameter** | **MEAN** |  | **S.D.** | **N** | **MEAN** |  | **S.D.** | **N** | **MEAN** |  | **S.D.** | **N** | **MEAN** |  | **S.D.** | **N** | **MEAN** |  | **S.D.** | **N** | **MEAN** |  | **S.D.** | **N** | **MEAN** |  | **S.D.** | **N** | **MEAN** |  | **S.D.** | **N** | **MEAN** |  | **S.D.** | **N** | **MEAN** |  | **S.D.** | **N** |
| **Leukocytes(10^3^ cells/μL)** | 2.874 | ± | 1.038 | 5 | 4.786 | ± | 1.415 | 5 | 4.278 | ± | 1.196 | 5 | 4.632 | ± | 1.148 | 5 | 4.816 | ± | 2.100 | 5 | 4.506 | ± | 1.808 | 5 | 4.100 | ± | 1.272 | 5 | 4.478 | ± | 0.805 | 5 | 2.056 | ± | 0.385 | 5 | 2.300 | ± | 0.417 | 5 |
| **Erythrocytes(10^6^ cells/μL)** | 8.608 | ± | 0.393 | 5 | 8.570 | ± | 0.245 | 5 | ***8.196** | **±** | **0.197** | **5** | ***7.978** | **±** | **0.140** | **5** | ****7.848** | **±** | **0.209** | **5** | 9.032 | ± | 0.512 | 5 | 8.420 | ± | 0.612 | 5 | ***8.102** | **±** | **0.190** | **5** | ***7.97** | **±** | **0.571** | **5** | ***7.954** | **±** | **0.143** | **5** |
| **Hemoglobin(g/dL)** | 13.74 | ± | 0.71 | 5 | 13.06 | ± | 0.65 | 5 | ***12.84** | **±** | **0.34** | **5** | ****12.18** | **±** | **0.42** | **5** | ****12.22** | **±** | **0.26** | **5** | 14.38 | ± | 0.76 | 5 | 13.12 | ± | 1.10 | 5 | 13.12 | ± | 0.268 | 5 | ***12.66** | **±** | **0.56** | **5** | ***12.7** | **±** | **0.67** | **5** |
| **Hematocrit(%)** | 44.88 | ± | 1.79 | 5 | 43.00 | ± | 1.67 | 5 | ***41.32** | **±** | **0.91** | **5** | ****40.06** | **±** | **1.59** | **5** | *****39.7** | **±** | **1.29** | **5** | 46.26 | ± | 2.07 | 5 | 43.16 | ± | 2.22 | 5 | ****42.76** | **±** | **0.416** | **5** | *****40.54** | **±** | **1.70** | **5** | *****40.2** | **±** | **1.37** | **5** |
| **MCV(fL)** | 52.20 | ± | 1.02 | 5 | 50.18 | ± | 1.00 | 5 | 49.74 | ± | 1.18 | 5 | 50.22 | ± | 1.79 | 5 | 50.60 | ± | 1.02 | 5 | 51.24 | ± | 1.38 | 5 | 51.30 | ± | 1.52 | 5 | 52.18 | ± | 1.12 | 5 | 50.96 | ± | 2.45 | 5 | 50.54 | ± | 1.10 | 5 |
| **MCH(pg)** | 16.00 | ± | 0.43 | 5 | 15.26 | ± | 0.57 | 5 | 15.44 | ± | 0.39 | 5 | 15.28 | ± | 0.41 | 5 | 15.58 | ± | 0.18 | 5 | 15.92 | ± | 0.53 | 5 | 15.60 | ± | 0.47 | 5 | 16.14 | ± | 0.52 | 5 | 15.92 | ± | 0.73 | 5 | 15.96 | ± | 0.61 | 5 |
| **MCHC(g/dL)** | 30.62 | ± | 0.55 | 5 | 30.38 | ± | 0.60 | 5 | 31.08 | ± | 0.46 | 5 | 30.44 | ± | 0.36 | 5 | 30.80 | ± | 0.60 | 5 | 31.08 | ± | 0.35 | 5 | 30.38 | ± | 1.32 | 5 | 30.96 | ± | 0.62 | 5 | 31.26 | ± | 0.37 | 5 | 31.60 | ± | 1.03 | 5 |
| **Platelets(10^3^ cells/μL)** | 1277.2 | ± | 51.8 | 5 | 1535.0 | ± | 199.4 | 5 | 1216.6 | ± | 166.6 | 5 | 1538.4 | ± | 214.5 | 5 | 1314.6 | ± | 140.9 | 5 | 1179.8 | ± | 190.3 | 5 | 1122.6 | ± | 246.2 | 5 | 1242.2 | ± | 96.6 | 5 | 874.6 | ± | 206.5 | 5 | 839.4 | ± | 111.5 | 5 |
| **Neutrophils(10^3^ cells/μL)** | 0.484 | ± | 0.153 | 5 | ***2.590** | **±** | **0.888** | **5** | ***2.994** | **±** | **1.086** | **5** | ***2.942** | **±** | **0.560** | **5** | ****3.332** | **±** | **1.834** | **5** | 0.746 | ± | 0.256 | 5 | 1.440 | ± | 0.545 | 5 | *****2.834** | **±** | **0.930** | **5** | 0.782 | ± | 0.070 | 5 | 1.254 | ± | 0.513 | 5 |
| **Neutrophils(%)** | 17.48 | ± | 4.13 | 5 | *****53.8** | **±** | **7.29** | **5** | *****68.96** | **±** | **5.84** | **5** | *****64.84** | **±** | **10.17** | **5** | *****65.22** | **±** | **13.79** | **5** | 17.34 | ± | 5.90 | 5 | 37.22 | ± | 13.98 | 5 | *****61.86** | **±** | **10.19** | **5** | 36.38 | ± | 8.05 | 5 | *****52.84** | **±** | **12.24** | **5** |
| **Lymphocytes(10^3^ cells/μL)** | 2.461 | ± | 0.600 | 5 | 2.022 | ± | 0.619 | 5 | ****1.066** | **±** | **0.192** | **5** | ***1.268** | **±** | **0.519** | **5** | ****1.302** | **±** | **0.267** | **5** | 3.550 | ± | 1.606 | 5 | 2.446 | ± | 1.385 | 5 | ***1.382** | **±** | **0.521** | **5** | ****1.102** | **±** | **0.432** | **5** | ****0.858** | **±** | **0.096** | **5** |
| **Lymphocytes(%)** | 76.66 | ± | 3.56 | 5 | *****42.44** | **±** | **7.37** | **5** | *****26.06** | **±** | **6.02** | **5** | *****30.7** | **±** | **8.42** | **5** | *****31** | **±** | **13.75** | **5** | 77.86 | ± | 6.43 | 5 | 57.64 | ± | 15.31 | 5 | *****32.6** | **±** | **8.753** | **5** | ***54.68** | **±** | **8.92** | **5** | *****38.78** | **±** | **9.99** | **5** |
| **Monocytes(10^3^ cells/μL)** | 0.044 | ± | 0.028 | 5 | ***0.012** | **±** | **0.008** | **5** | ***0.01** | **±** | **0.000** | **5** | ***0.01** | **±** | **0.007** | **5** | ****0.008** | **±** | **0.008** | **5** | 0.062 | ± | 0.022 | 5 | *****0.002** | **±** | **0.004** | **5** | *****0.012** | **±** | **0.008** | **5** | *****0.002** | **±** | **0.004** | **5** | *****0.006** | **±** | **0.005** | **5** |
| **Monocytes(%)** | 1.600 | ± | 0.964 | 5 | ***0.24** | **±** | **0.114** | **5** | ***0.24** | **±** | **0.055** | **5** | ***0.22** | **±** | **0.110** | **5** | ****0.14** | **±** | **0.114** | **5** | 1.14 | ± | 0.167 | 5 | *****0.100** | **±** | **0.071** | **5** | *****0.18** | **±** | **0.130** | **5** | *****0.240** | **±** | **0.114** | **5** | *****0.260** | **±** | **0.152** | **5** |
| **Eosinophils(10^3^ cells/μL)** | 0.108 | ± | 0.087 | 5 | 0.054 | ± | 0.034 | 5 | 0.088 | ± | 0.061 | 5 | 0.102 | ± | 0.102 | 5 | 0.092 | ± | 0.059 | 5 | 0.112 | ± | 0.064 | 5 | 0.076 | ± | 0.067 | 5 | 0.094 | ± | 0.060 | 5 | 0.030 | ± | 0.021 | 5 | 0.056 | ± | 0.015 | 5 |
| **Eosinophils(%)** | 3.300 | ± | 1.962 | 5 | 1.120 | ± | 0.476 | 5 | 2.000 | ± | 0.995 | 5 | 1.960 | ± | 1.811 | 5 | 1.720 | ± | 0.657 | 5 | 2.820 | ± | 1.720 | 5 | 1.960 | ± | 1.498 | 5 | 2.1 | ± | 1.284 | 5 | 2.380 | ± | 2.017 | 5 | 2.440 | ± | 0.773 | 5 |
| **Basophils(10^3^ cells/μL)** | 0.006 | ± | 0.005 | 5 | 0.006 | ± | 0.005 | 5 | 0.010 | ± | 0.000 | 5 | 0.010 | ± | 0.007 | 5 | 0.008 | ± | 0.008 | 5 | 0.012 | ± | 0.004 | 5 | 0.010 | ± | 0.007 | 5 | 0.014 | ± | 0.005 | 5 | 0.008 | ± | 0.004 | 5 | 0.014 | ± | 0.005 | 5 |
| **Basophils(%)** | 0.280 | ± | 0.303 | 5 | 0.200 | ± | 0.071 | 5 | 0.200 | ± | 0.071 | 5 | 0.180 | ± | 0.110 | 5 | 0.180 | ± | 0.084 | 5 | 0.320 | ± | 0.110 | 5 | 0.300 | ± | 0.071 | 5 | 0.28 | ± | 0.083 | 5 | 0.540 | ± | 0.391 | 5 | 0.520 | ± | 0.217 | 5 |
| **Reticulocyte(10^9^ cells/μL)** | 369.2 | ± | 47.0 | 5 | 330.8 | ± | 50.8 | 5 | *****175.6** | **±** | **49.4** | **5** | ***279.1** | **±** | **43.3** | **5** | ****231.9** | **±** | **33.0** | **5** | 584.5 | ± | 93.7 | 5 | 469.1 | ± | 77.9 | 5 | *****341.0** | **±** | **80.261** | **5** | *****155.9** | **±** | **51.1** | **5** | *****156.3** | **±** | **50.6** | **5** |
| **Reticulocyte(%)** | 4.288 | ± | 0.500 | 5 | 3.870 | ± | 0.651 | 5 | *****2.078** | **±** | **0.575** | **5** | **3.496** | **±** | **0.525** | **5** | ***2.966** | **±** | **0.496** | **5** | 6.500 | ± | 1.173 | 5 | 5.554 | ± | 0.646 | 5 | ****4.144** | **±** | **1.003** | **5** | *****1.988** | **±** | **0.774** | **5** | *****1.974** | **±** | **0.678** | **5** |
| **S.D., Standard Deviation** | | | | | | | | | | | | | | | | | | | | | | | | | | | | | | | | | | | | | | | | |
| **N, Number of Animals** | | | | | | | | | | | | | | | | | | | | | | | | | | | | | | | | | | | | | | | | |
| ***, Significantly different from the control group; p<0.05** | | | | | | | | | | | | | | | | | | | | | | | | | | | | | | | | | | | | | | | | |
| ****, Significantly different from the control group; p<0.01** | | | | | | | | | | | | | | | | | | | | | | | | | | | | | | | | | | | | | | | | |
| *****, Significantly different from the control group; p<0.001** | | | | | | | | | | | | | | | | | | | | | | | | | | | | | | | | | | | | | | | | |

**Supplementary data 3. Group summary of hematology in study #1 (2 day post second injection)** Levels of indicated parameters were analyzed using a complete blood count (CBC) on necropsy day (2 dpsi). The data were presented with Mean ± SD (n=5), and the statistically significant differences between the negative control and each test group were analyzed using the Kruskal-Wallis test followed by Bonferroni's post hoc test (*p<0.05, **p<0.01, ***p<0.001).

| **<Study #1> Group Summary of Serum Blood Chemistry (2 day post second injection)** | | | | | | | | | | | | | | | | | | | | | | | | | | | | | | | | | | | | | | | | |
| --- | --- | --- | --- | --- | --- | --- | --- | --- | --- | --- | --- | --- | --- | --- | --- | --- | --- | --- | --- | --- | --- | --- | --- | --- | --- | --- | --- | --- | --- | --- | --- | --- | --- | --- | --- | --- | --- | --- | --- | --- |
| **Sex** | **Male** | | | | | | | | | | | | | | | | | | | | **Female** | | | | | | | | | | | | | | | | | | | |
| **Test Item** | **D-PBS** | | | | **CUK3-1/LNP-A** | | | | **CUK3-1/LNP-B** | | | | **CUK3-1/LNP-C** | | | | **CUK3-1/LNP-D** | | | | **D-PBS** | | | | **CUK3-1/LNP-A** | | | | **CUK3-1/LNP-B** | | | | **CUK3-1/LNP-C** | | | | **CUK3-1/LNP-D** | | | |
| **mRNA Dosage (μg/head)** | **0** | | | | **50** | | | | **50** | | | | **50** | | | | **50** | | | | **0** | | | | **50** | | | | **50** | | | | **50** | | | | **50** | | | |
| **Parameter** | **MEAN** |  | **S.D.** | **N** | **MEAN** |  | **S.D.** | **N** | **MEAN** |  | **S.D.** | **N** | **MEAN** |  | **S.D.** | **N** | **MEAN** |  | **S.D.** | **N** | **MEAN** |  | **S.D.** | **N** | **MEAN** |  | **S.D.** | **N** | **MEAN** |  | **S.D.** | **N** | **MEAN** |  | **S.D.** | **N** | **MEAN** |  | **S.D.** | **N** |
| **Calcium (mg/dL)** | 8.92 | ± | 0.20 | 5 | 9.28 | ± | 0.19 | 5 | 9.2 | ± | 0.16 | 5 | 9.18 | ± | 0.15 | 5 | 9.12 | ± | 0.32 | 5 | 9.08 | ± | 0.23 | 5 | 9.14 | ± | 0.35 | 5 | 9.46 | ± | 0.21 | 5 | 9.28 | ± | 0.20 | 5 | 8.98 | ± | 0.21 | 5 |
| **Inorganic Phosphorus (mg/dL)** | 7.12 | ± | 0.77 | 5 | 7 | ± | 0.62 | 5 | 7.16 | ± | 0.72 | 5 | 7.66 | ± | 0.93 | 5 | 7.06 | ± | 0.72 | 5 | 7.34 | ± | 0.52 | 5 | 6.96 | ± | 1.31 | 5 | 7.34 | ± | 0.69 | 5 | 5.76 | ± | 1.07 | 5 | 6.78 | ± | 0.56 | 5 |
| **Glucose (mg/L)** | 113.4 | ± | 27.0 | 5 | 80.4 | ± | 22.8 | 5 | 71.8 | ± | 21.3 | 5 | 82 | ± | 17.8 | 5 | *63 | ± | 12.4 | 5 | 110.2 | ± | 43.1 | 5 | 75.2 | ± | 11.7 | 5 | 77.6 | ± | 15.0 | 5 | 66.6 | ± | 13.8 | 5 | 90 | ± | 17.0 | 5 |
| **BUN (mg/dL)** | 20.94 | ± | 4.34 | 5 | 20.5 | ± | 4.7 | 5 | 22.08 | ± | 6.06 | 5 | 22.08 | ± | 5.51 | 5 | 18.24 | ± | 4.38 | 5 | 18.04 | ± | 4.58 | 5 | 17.04 | ± | 1.75 | 5 | 16.8 | ± | 4.33 | 5 | 13.8 | ± | 1.75 | 5 | 16.3 | ± | 3.24 | 5 |
| **Creatinine (mg/dL)** | 0.296 | ± | 0.031 | 5 | 0.288 | ± | 0.050 | 5 | 0.308 | ± | 0.023 | 5 | 0.284 | ± | 0.038 | 5 | 0.284 | ± | 0.013 | 5 | 0.3 | ± | 0.04 | 5 | 0.34 | ± | 0.02 | 5 | 0.282 | ± | 0.04 | 5 | 0.286 | ± | 0.01 | 5 | 0.304 | ± | 0.03 | 5 |
| **Cholesterol (mg/dL)** | 153.6 | ± | 31.1 | 5 | 173.8 | ± | 8.8 | 5 | 140.2 | ± | 16.8 | 5 | 165.2 | ± | 33.4 | 5 | 133 | ± | 17.8 | 5 | 98.2 | ± | 20.3 | 5 | 106.6 | ± | 13.27 | 5 | 93.6 | ± | 22.3 | 5 | 106 | ± | 13.4 | 5 | 106.6 | ± | 14.7 | 5 |
| **Total Protein (g/dL)** | 4.86 | ± | 0.32 | 5 | 4.88 | ± | 0.11 | 5 | 4.52 | ± | 0.13 | 5 | 4.84 | ± | 0.19 | 5 | 4.68 | ± | 0.19 | 5 | 4.96 | ± | 0.08 | 5 | 4.92 | ± | 0.41 | 5 | 4.82 | ± | 0.17 | 5 | 4.52 | ± | 0.15 | 5 | ***4.48** | **±** | **0.10** | **5** |
| **Albumin (g/dL)** | 1.84 | ± | 0.11 | 5 | *****1.6** | **±** | **0.07** | **5** | *****1.4** | **±** | **0** | **5** | *****1.56** | **±** | **0.05** | 5 | *****1.38** | **±** | **0.04** | **5** | 1.86 | ± | 0.05 | 5 | ****1.62** | **±** | **0.16** | **5** | *****1.54** | **±** | **0.05** | **5** | *****1.44** | **±** | **0.05** | **5** | *****1.52** | **±** | **0.04** | **5** |
| **Total Bilirubin (mg/dL)** | 0.138 | ± | 0.026 | 5 | *****0.072** | **±** | **0.013** | **5** | ****0.076** | **±** | **0.015** | **5** | ****0.074** | **±** | **0.018** | 5 | *****0.058** | **±** | **0.024** | **5** | 0.102 | ± | 0.02 | 5 | ****0.04** | **±** | **0.023** | **5** | ***0.052** | **±** | **0.022** | **5** | ****0.042** | **±** | **0.013** | **5** | ****0.05** | **±** | **0.018** | **5** |
| **ALP (IU/L)** | 197.8 | ± | 24.3 | 5 | *****104.6** | **±** | **35.8** | **5** | *****56** | **±** | **8.6** | **5** | *****89.6** | **±** | **14.01** | 5 | *****60.6** | **±** | **15.53** | **5** | 351.4 | ± | 85.7 | 5 | *****117.2** | **±** | **32.8** | **5** | *****85** | **±** | **6.6** | **5** | *****83.6** | **±** | **13.2** | **5** | *****86** | **±** | **15.03** | **5** |
| **AST (IU/L)** | 68.8 | ± | 19.7 | 5 | 73.8 | ± | 11.5 | 5 | *****117.2** | **±** | **7.3** | **5** | 78.6 | ± | 5.02 | 5 | *****114.6** | **±** | **16.28** | **5** | 77.4 | ± | 13.18 | 5 | 86.2 | ± | 12.4 | 5 | *****121.2** | **±** | **12.8** | **5** | 95.8 | ± | 17.9 | 5 | ****115.4** | **±** | **20.2** | **5** |
| **ALT (IU/L)** | 29.0 | ± | 6.1 | 5 | 26.0 | ± | 3.4 | 5 | 31.4 | ± | 5.6 | 5 | 24 | ± | 2.4 | 5 | 31.2 | ± | 6.01 | 5 | 30.6 | ± | 8.64 | 5 | 24.2 | ± | 2.7 | 5 | 24.6 | ± | 5.3 | 5 | 34.6 | ± | 8.7 | 5 | 34.2 | ± | 13.4 | 5 |
| **Triglycerides (mg/dL)** | 155.6 | ± | 89.2 | 5 | 155.6 | ± | 34.9 | 5 | 171.6 | ± | 72.7 | 5 | 157.8 | ± | 28.7 | 5 | 100.8 | ± | 20.1 | 5 | 121.2 | ± | 36.6 | 5 | 194.8 | ± | 57.9 | 5 | 132.8 | ± | 76.9 | 5 | 143.8 | ± | 47.7 | 5 | 127 | ± | 34.33 | 5 |
| **HDL cholesterol (mg/dL)** | 65.0 | ± | 10.1 | 5 | 64.8 | ± | 5.7 | 5 | ***51** | **±** | **2.9** | **5** | 56.4 | ± | 9.2 | 5 | ****46.8** | **±** | **3.9** | **5** | 50 | ± | 10.8 | 5 | 46.8 | ± | 3.8 | 5 | 40 | ± | 9.0 | 5 | 40.8 | ± | 3.1 | 5 | 45.2 | ± | 5.31 | 5 |
| **LDL cholesterol (mg/dL)** | 8.6 | ± | 4.5 | 5 | 14.4 | ± | 4.8 | 5 | 12.6 | ± | 3.6 | 5 | ***16.8** | **±** | **2.6** | **5** | 13 | ± | 2.7 | 5 | 4.6 | ± | 1.14 | 5 | 8.2 | ± | 2.77 | 5 | 8 | ± | 2.34 | 5 | ***11** | **±** | **4.30** | **5** | ***11** | **±** | **2.12** | **5** |
| **Sodium (mmol/L)** | 150.8 | ± | 0.4 | 5 | 149.4 | ± | 1.9 | 5 | 149.8 | ± | 2.4 | 5 | 148.6 | ± | 1.7 | 5 | 148.6 | ± | 1.5 | 5 | 148.4 | ± | 1.51 | 5 | 148.4 | ± | 0.89 | 5 | 147.4 | ± | 1.81 | 5 | 148 | ± | 1.5 | 5 | 148 | ± | 2.12 | 5 |
| **Potassium (mmol/L)** | 5.01 | ± | 0.24 | 5 | 5.42 | ± | 0.17 | 5 | 5.36 | ± | 0.23 | 5 | 5.28 | ± | 0.36 | 5 | 5.22 | ± | 0.13 | 5 | 4.98 | ± | 0.60 | 5 | 5.32 | ± | 0.66 | 5 | 5.46 | ± | 0.32 | 5 | 4.88 | ± | 0.47 | 5 | 5.38 | ± | 0.35 | 5 |
| **Chloride (mmol/L)** | 111.2 | ± | 1.3 | 5 | 108.4 | ± | 2.7 | 5 | 108.2 | ± | 2.0 | 5 | 107.4 | ± | 2.4 | 5 | 107.2 | ± | 1.5 | 5 | 108.8 | ± | 2.38 | 5 | 108 | ± | 1.22 | 5 | 106.4 | ± | 2.51 | 5 | 108.2 | ± | 2.16 | 5 | 108 | ± | 2.91 | 5 |
| **Cardiac Troponin-I (pg/mL)** | 10.11 | ± | 13.96 | 5 | ***585.9** | **±** | **492.9** | **5** | ****1019.4** | **±** | **464.07** | **5** | ****1149.2** | **±** | **726.21** | **5** | ****1501.8** | **±** | **474.49** | **5** | 7.59 | ± | 14.3 | 5 | ***398.6** | **±** | **226.5** | **5** | ****1146.2** | **±** | **345.4** | **5** | ****1441.1** | **±** | **701.0** | **5** | ****1365.4** | **±** | **631.4** | **5** |
| **NT-proBNP (pg/mL)** | 352.8 | ± | 100.7 |  | ****3674.0** | **±** | **653.1** | **5** | *****6791.0** | **±** | **910.0** | **5** | *****5982.9** | **±** | **625.0** | **5** | *****7212.7** | **±** | **470.9** | **5** | 448.0 | ± | 172.3 | 5 | ****4592.8** | **±** | **1190.0** | **5** | *****6074.7** | **±** | **746.1** | **5** | *****6491.2** | **±** | **1245.0** | **5** | *****6516.2** | **±** | **567.1** | **5** |
| **S.D., Standard Deviation** | | | | | | | | | | | | | | | | | | | | | | | | | | | | | | | | | | | | | | | | |
| **N, Number of Animals** | | | | | | | | | | | | | | | | | | | | | | | | | | | | | | | | | | | | | | | | |
| ***, Significantly different from the control group; p<0.05** | | | | | | | | | | | | | | | | | | | | | | | | | | | | | | | | | | | | | | | | |
| ****, Significantly different from the control group; p<0.01** | | | | | | | | | | | | | | | | | | | | | | | | | | | | | | | | | | | | | | | | |
| *****, Significantly different from the control group; p<0.001** | | | | | | | | | | | | | | | | | | | | | | | | | | | | | | | | | | | | | | | | |

**Supplementary data 4. Group summary of blood chemistry in study #1 (2 day post second injection)** Levels of indicated parameters were analyzed using serum blood chemistry on necropsy day (2 dpsi). The data were presented with Mean ± SD (n=5), and the statistically significant differences between the negative control and each test group were analyzed using the Kruskal-Wallis test followed by the Bonferroni post hoc test (*p<0.05, **p<0.01, ***p<0.001).

| **<Study #1> Group Summary Organ Weight (Unit: g, % of body weight, 2 day post second injection)** | | | | | | | | | | | | | | | | | | | | | | | | | | | | | | | | | | | | | | | | |
| --- | --- | --- | --- | --- | --- | --- | --- | --- | --- | --- | --- | --- | --- | --- | --- | --- | --- | --- | --- | --- | --- | --- | --- | --- | --- | --- | --- | --- | --- | --- | --- | --- | --- | --- | --- | --- | --- | --- | --- | --- |
| **Sex** | **Male** | | | | | | | | | | | | | | | | | | | | **Female** | | | | | | | | | | | | | | | | | | | |
| **Test Item** | **D-PBS** | | | | **CUK3-1/LNP-A** | | | | **CUK3-1/LNP-B** | | | | **CUK3-1/LNP-C** | | | | **CUK3-1/LNP-D** | | | | **D-PBS** | | | | **CUK3-1/LNP-A** | | | | **CUK3-1/LNP-B** | | | | **CUK3-1/LNP-C** | | | | **CUK3-1/LNP-D** | | | |
| **mRNA Dosage (μg/head)** | **0** | | | | **50** | | | | **50** | | | | **50** | | | | **50** | | | | **0** | | | | **50** | | | | **50** | | | | **50** | | | | **50** | | | |
| **Parameter** | **MEAN** |  | **S.D.** | **N** | **MEAN** |  | **S.D.** | **N** | **MEAN** |  | **S.D.** | **N** | **MEAN** |  | **S.D.** | **N** | **MEAN** |  | **S.D.** | **N** | **MEAN** |  | **S.D.** | **N** | **MEAN** |  | **S.D.** | **N** | **MEAN** |  | **S.D.** | **N** | **MEAN** |  | **S.D.** | **N** | **MEAN** |  | **S.D.** | **N** |
| **Necropsy B.W** | 35.15 | ± | 1.94 | 5 | 34.43 | ± | 1.99 | 5 | 34.48 | ± | 2.20 | 5 | 34.07 | ± | 1.37 | 5 | 34.19 | ± | 2.39 | 5 | 25.67 | ± | 1.727 | 5 | 26.16 | ± | 1.182 | 5 | 26.62 | ± | 1.585 | 5 | 25.25 | ± | 1.679 | 5 | 25.22 | ± | 0.796 | 5 |
| **Spleen** | 0.295 | ± | 0.034 | 5 | 0.414 | ± | 0.083 | 5 | ****0.508** | **±** | **0.111** | **5** | ****0.496** | **±** | **0.087** | **5** | *****0.583** | **±** | **0.057** | **5** | 0.459 | ± | 0.038 | 5 | 0.573 | ± | 0.066 | 5 | ****0.702** | **±** | **0.108** | **5** | ****0.689** | **±** | **0.095** | **5** | ****0.721** | **±** | **0.077** | **5** |
| **Liver** | 3.962 | ± | 0.198 | 5 | 4.315 | ± | 0.268 | 5 | *****4.930** | **±** | **0.143** | **5** | *****4.760** | **±** | **0.250** | **5** | *****4.958** | **±** | **0.277** | **5** | 4.080 | ± | 0.235 | 5 | 4.477 | ± | 0.334 | 5 | ****4.966** | **±** | **0.390** | **5** | *****5.117** | **±** | **0.163** | **5** | ****5.021** | **±** | **0.273** | **5** |
| **Kidney (R)** | 0.690 | ± | 0.063 | 5 | 0.707 | ± | 0.049 | 5 | 0.679 | ± | 0.083 | 5 | 0.755 | ± | 0.084 | 5 | 0.775 | ± | 0.084 | 5 | 0.627 | ± | 0.070 | 5 | 0.575 | ± | 0.049 | 5 | 0.620 | ± | 0.048 | 5 | 0.639 | ± | 0.040 | 5 | 0.626 | ± | 0.038 | 5 |
| **Kidney (L)** | 0.670 | ± | 0.068 | 5 | 0.709 | ± | 0.044 | 5 | 0.657 | ± | 0.082 | 5 | 0.714 | ± | 0.074 | 5 | 0.754 | ± | 0.113 | 5 | 0.622 | ± | 0.052 | 5 | 0.584 | ± | 0.066 | 5 | 0.615 | ± | 0.028 | 5 | 0.608 | ± | 0.023 | 5 | 0.603 | ± | 0.055 | 5 |
| **Adrenal gland (R)** | 0.0067 | ± | 0.0017 | 5 | 0.0097 | ± | 0.0024 | 5 | 0.0082 | ± | 0.0001 | 5 | 0.0101 | ± | 0.0034 | 5 | 0.0084 | ± | 0.0016 | 5 | 0.0157 | ± | 0.0026 | 5 | 0.0181 | ± | 0.0047 | 5 | 0.0163 | ± | 0.0043 | 5 | 0.0182 | ± | 0.0025 | 5 | 0.0167 | ± | 0.0040 | 5 |
| **Adrenal gland (L)** | 0.0079 | ± | 0.0020 | 5 | 0.0095 | ± | 0.0020 | 5 | 0.0093 | ± | 0.0020 | 5 | 0.0083 | ± | 0.0020 | 5 | 0.0088 | ± | 0.0019 | 5 | 0.0170 | ± | 0.0037 | 5 | 0.0193 | ± | 0.0028 | 5 | 0.0182 | ± | 0.0057 | 5 | 0.0198 | ± | 0.0014 | 5 | 0.0179 | ± | 0.0028 | 5 |
| **Prostate/Uterus** | 0.902 | ± | 0.105 | 5 | 0.963 | ± | 0.179 | 5 | 0.899 | ± | 0.136 | 5 | 0.970 | ± | 0.104 | 5 | 0.970 | ± | 0.144 | 5 | 0.677 | ± | 0.164 | 5 | 0.500 | ± | 0.202 | 5 | 0.696 | ± | 0.272 | 5 | 0.740 | ± | 0.182 | 5 | 1.964 | ± | 0.204 | 5 |
| **Testis/Ovary (R)** | 0.317 | ± | 0.031 | 5 | 0.354 | ± | 0.052 | 5 | 0.332 | ± | 0.050 | 5 | 0.344 | ± | 0.037 | 5 | 0.337 | ± | 0.021 | 5 | 0.024 | ± | 0.004 | 5 | 0.024 | ± | 0.004 | 5 | 0.024 | ± | 0.004 | 5 | 0.024 | ± | 0.012 | 5 | 0.340 | ± | 0.033 | 5 |
| **Testis/Ovary (L)** | 0.314 | ± | 0.043 | 5 | 0.354 | ± | 0.066 | 5 | 0.334 | ± | 0.047 | 5 | 0.331 | ± | 0.036 | 5 | 0.320 | ± | 0.024 | 5 | 0.025 | ± | 0.005 | 5 | 0.018 | ± | 0.010 | 5 | 0.024 | ± | 0.002 | 5 | 0.418 | ± | 0.036 | 5 | 0.328 | ± | 0.030 | 5 |
| **Thymus** | 0.187 | ± | 0.022 | 5 | 0.145 | ± | 0.013 | 5 | ***0.122** | **±** | **0.025** | **5** | ***0.136** | **±** | **0.009** | **5** | ****0.107** | **±** | **0.012** | **5** | 0.286 | ± | 0.031 | 5 | 0.217 | ± | 0.055 | 5 | ****0.189** | **±** | **0.023** | **5** | ****0.187** | **±** | **0.026** | **5** | ***0.195** | **±** | **0.028** | **5** |
| **Heart** | 0.451 | ± | 0.030 | 5 | 0.439 | ± | 0.030 | 5 | 0.429 | ± | 0.019 | 5 | 0.427 | ± | 0.036 | 5 | 0.456 | ± | 0.034 | 5 | 0.484 | ± | 0.031 | 5 | 0.444 | ± | 0.054 | 5 | 0.438 | ± | 0.022 | 5 | 0.478 | ± | 0.023 | 5 | 0.464 | ± | 0.029 | 5 |
| **Lung** | 0.521 | ± | 0.028 | 5 | 0.560 | ± | 0.040 | 5 | 0.554 | ± | 0.028 | 5 | 0.534 | ± | 0.030 | 5 | 0.546 | ± | 0.036 | 5 | 0.604 | ± | 0.048 | 5 | 0.607 | ± | 0.032 | 5 | 0.573 | ± | 0.048 | 5 | 0.648 | ± | 0.066 | 5 | 0.658 | ± | 0.026 | 5 |
| **Brain** | 1.372 | ± | 0.162 | 5 | 1.450 | ± | 0.089 | 5 | 1.454 | ± | 0.114 | 5 | 1.417 | ± | 0.059 | 5 | 1.486 | ± | 0.102 | 5 | 1.837 | ± | 0.106 | 5 | 1.805 | ± | 0.086 | 5 | 1.788 | ± | 0.111 | 5 | 1.879 | ± | 0.069 | 5 | 1.863 | ± | 0.127 | 5 |
| **S.D., Standard Deviation** | | | | | | | | | | | | | | | | | | | | | | | | | | | | | | | | | | | | | | | | |
| **N, Number of Animals** | | | | | | | | | | | | | | | | | | | | | | | | | | | | | | | | | | | | | | | | |
| ***, Significantly different from the control group; p<0.05** | | | | | | | | | | | | | | | | | | | | | | | | | | | | | | | | | | | | | | | | |
| ****, Significantly different from the control group; p<0.01** | | | | | | | | | | | | | | | | | | | | | | | | | | | | | | | | | | | | | | | | |
| *****, Significantly different from the control group; p<0.001** | | | | | | | | | | | | | | | | | | | | | | | | | | | | | | | | | | | | | | | | |

**Supplementary data 5. Group summary of organ weight relative to body weight in study #1 (2 day post second injection)** The absolute weight of indicated organs was determined on necropsy day (2 dpsi), and then converted to relative weight (% of body weight). The data, presented as Mean ± SD (n=5), showed statistically significant differences between the negative control and each test group, analyzed using the Kruskal-Wallis test and the Bonferroni post hoc test (*p<0.05, **p<0.01, ***p<0.001).

| **<Study #1> Group Summary of Histopathological Findings (2 day post second injection)** | | | | | | | | | | | |
| --- | --- | --- | --- | --- | --- | --- | --- | --- | --- | --- | --- |
| **Sex** | **Male** | | | | | **Female** | | | | | |
| **Test Item** | **D-PBS** | **CUK3-1/LNP-A** | **CUK3-1/LNP-B** | **CUK3-1/LNP-C** | **CUK3-1/LNP-D** | **D-PBS** | **CUK3-1/LNP-A** | **CUK3-1/LNP-B** | **CUK3-1/LNP-C** | **CUK3-1/LNP-D** |  |
| **mRNA Dosage (㎍/head)** | **0** | **50** | **50** | **50** | **50** | **0** | **50** | **50** | **50** | **50** |  |
| **Tissue and observed content** | **Incidence (a/b)** | | | | | | | | | | |
| **Injection site/Quadriceps muscle** |  |  |  |  |  |  |  |  |  |  |  |
| No abnormalities detected | 5/5 |  |  |  |  | 5/5 |  |  |  |  |  |
| Inflammation, acute (minimal) |  | 2/5 |  |  |  |  | 3/5 |  |  | 2/5 |  |
| Inflammation, acute (mild) |  | 3/5 | 3/5 | 3/5 | 2/5 |  | 2/5 | 1/5 | 5/5 | 2/5 |  |
| Inflammation, acute (moderate) |  |  | 2/5 | 2/5 | 3/5 |  |  | 4/5 |  | 1/5 |  |
| Degeneration, myofiber (minimal) |  |  |  |  |  |  |  | 1/5 |  |  |  |
| Necrosis, myofiber (minimal) |  | 1/5 |  |  |  |  |  |  | 1/5 |  |  |
| Degeneration & necrosis, myofiber (minimal) |  |  |  |  | 1/5 |  |  |  | 1/5 |  |  |
| Degeneration & necrosis, myofiber (mild) |  |  |  |  |  |  |  |  | 2/5 |  |  |
| **Bone marrow/Femur** |  |  |  |  |  |  |  |  |  |  |  |
| No abnormalities detected | 5/5 | 1/5 |  |  |  | 5/5 | 3/5 |  |  |  |  |
| Cellularity, decreased, erythroid cell (minimal) |  | 3/5 |  |  |  |  | 2/5 |  |  |  |  |
| Cellularity, decreased, erythroid cell (mild) |  | 1/5 | 1/5 | 5/5 | 1/5 |  |  | 3/5 |  | 3/5 |  |
| Cellularity, decreased, erythroid cell (moderate) |  |  | 4/5 |  | 4/5 |  |  | 2/5 | 5/5 | 2/5 |  |
| **Spleen** |  |  |  |  |  |  |  |  |  |  |  |
| No abnormalities detected | 5/5 | 2/5 |  |  | 1/5 | 5/5 | 5/5 |  |  |  |  |
| Cellularity, increased, lymphocyte, white pulp (minimal) |  | 1/5 | 3/5 | 1/5 | 3/5 |  |  | 3/5 | 2/5 | 1/5 |  |
| Cellularity, increased, lymphocyte, white pulp (mild) |  |  |  |  | 1/5 |  |  |  | 2/5 | 1/5 |  |
| Infiltration, neutrophil, red pulp (minimal) |  | 2/5 | 2/5 | 3/5 | 2/5 |  |  | 2/5 |  | 1/5 |  |
| Infiltration, neutrophil, red pulp (mild) |  |  | 1/5 |  | 1/5 |  |  |  |  |  |  |
| Cellularity, decreased, erythroid cell, red pulp (minimal) |  |  | 2/5 | 1/5 |  |  |  | 1/5 | 2/5 | 3/5 |  |
| Cellularity, decreased, erythroid cell, red pulp (mild) |  |  | 3/5 | 1/5 | 3/5 |  |  | 1/5 | 2/5 | 1/5 |  |
| Cellularity, increased, megakaryocyte (minimal) |  | 1/5 | 2/5 | 4/5 | 2/5 |  |  | 2/5 | 2/5 | 2/5 |  |
| Cellularity, increased, megakaryocyte (mild) |  |  |  |  | 1/5 |  |  | 1/5 | 1/5 | 2/5 |  |
| Extramedullary hematopoiesis, increased, granulopoiesis (minimal) |  |  | 1/5 | 1/5 | 3/5 |  |  | 3/5 | 2/5 | 3/5 |  |
| **Thymus** |  |  |  |  |  |  |  |  |  |  |  |
| No abnormalities detected | 5/5 | 2/5 | 1/5 | 3/5 |  | 5/5 | 4/5 | 3/5 |  |  |  |
| Cortical atrophy (minimal) |  | 3/5 | 3/5 | 1/5 | 1/5 |  |  | 2/5 | 2/5 | 2/5 |  |
| Cortical atrophy (mild) |  |  | 1/5 |  | 3/5 |  |  |  | 2/5 | 1/5 |  |
| Cortical atrophy (moderate) |  |  |  |  |  |  |  |  | 1/5 |  |  |
| Tingible body macrophage, increased (minimal) |  |  | 3/5 | 1/5 | 3/5 |  | 1/5 | 2/5 | 4/5 | 5/5 |  |
| Tingible body macrophage, increased (mild) |  |  |  |  | 2/5 |  |  |  | 1/5 |  |  |
| **a, Number of observed animals.** | | | | | | | | | | |  |
| **b, Number of total animals** | | | | | | | | | | |  |

**Supplementary data 6. Group summary of histopathological findings in study #1 (2 days post second injection)** Histopathological changes induced by mRNA vaccine candidates in major organs (Liver, Lung, Kidney, Adrenal gland, Heart, Femur bone marrow, Quadriceps muscle, Testis/Ovary, Thymus and Spleen) were examined using H&E stain. The organs showing changes and the content observed were reported as incidences. The findings pertain to 2 days post second injection.

| **<Study #1> Group Summary of Hematology (14 day post second injection)^1^** | | | | | | | | | | | | | | | | | | | | | | | | | | | | | | | | | | | | | | | | | | | | | | |
| --- | --- | --- | --- | --- | --- | --- | --- | --- | --- | --- | --- | --- | --- | --- | --- | --- | --- | --- | --- | --- | --- | --- | --- | --- | --- | --- | --- | --- | --- | --- | --- | --- | --- | --- | --- | --- | --- | --- | --- | --- | --- | --- | --- | --- | --- | --- |
| **Sex** | **Male** | | | | | | | | | | | | | | | | | | | | | | **Female** | | | | | | | | | | | | | | | | | | | | | | | |
| **Test Item** | **D-PBS** | | | | **CUK3-1/LNP-A** | | | | **CUK3-1/LNP-B** | | | | **CUK3-1/LNP-C** | | | | | **CUK3-1/LNP-D** | | | | | **D-PBS** | | | | | **CUK3-1/LNP-A** | | | | | **CUK3-1/LNP-B** | | | | | **CUK3-1/LNP-C** | | | | | **CUK3-1/LNP-D** | | | |
| **mRNA Dosage (μg/head)** | **0** | | | | **50** | | | | **50** | | | | **50** | | | | | **50** | | | | | **0** | | | | | **50** | | | | | **50** | | | | | **50** | | | | | **50** | | | |
| **Parameter** | **MEAN** |  | **S.D.** | **N** | **MEAN** |  | **S.D.** | **N** | **MEAN** |  | **S.D.** | **N** | **MEAN** |  | **S.D.** | **N** | **MEAN** | |  | **S.D.** | **N** | **MEAN** | |  | **S.D.** | **N** | **MEAN** | |  | **S.D.** | **N** | **MEAN** | |  | **S.D.** | **N** | **MEAN** | |  | **S.D.** | **N** | **MEAN** | |  | **S.D.** | **N** |
| **Leukocytes(10^3^ cells/μL)** | 1.97 | ± | 0.77 | 5 | 4.19 | ± | 1.20 | 5 | 2.01 | **±** | 0.70 | 4 | 2.51 | ± | 1.38 | 5 | 3.15 | | ± | 2.71 | 5 | 1.98 | | ± | 0.53 | 5 | 4.95 | | ± | 1.57 | 5 | 4.55 | | ± | 0.75 | 5 | 3.70 | | ± | 1.40 | 5 | 3.54 | | ± | 2.21 | 5 |
| **Erythrocytes(10^6^ cells/μL)** | 8.65 | ± | 0.41 | 5 | 8.49 | ± | 0.44 | 5 | 8.54 | **±** | 0.24 | 4 | 8.58 | ± | 0.49 | 5 | 8.44 | | ± | 0.25 | 5 | 8.79 | | ± | 0.23 | 5 | 8.48 | | ± | 0.32 | 5 | 8.14 | | ± | 0.47 | 5 | 8.50 | | ± | 0.58 | 5 | 8.25 | | ± | 0.23 | 5 |
| **Hemoglobin(g/dL)** | 13.74 | ± | 0.73 | 5 | 13.04 | ± | 0.55 | 5 | 12.85 | **±** | 0.50 | 4 | 13.12 | ± | 0.47 | 5 | 12.90 | | ± | 0.49 | 5 | 13.86 | | ± | 0.63 | 5 | 13.26 | | ± | 0.53 | 5 | 13.08 | | ± | 0.85 | 5 | 13.38 | | ± | 0.60 | 5 | 12.92 | | ± | 0.79 | 5 |
| **Hematocrit(%)** | 44.50 | ± | 2.39 | 5 | 43.10 | ± | 1.65 | 5 | 42.85 | **±** | 1.63 | 4 | 43.66 | ± | 1.25 | 5 | 43.14 | | ± | 1.74 | 5 | 45.34 | | ± | 2.16 | 5 | 43.70 | | ± | 1.87 | 5 | 42.04 | | ± | 2.90 | 5 | 43.76 | | ± | 1.94 | 5 | 42.26 | | ± | 2.15 | 5 |
| **MCV(fL)** | 51.44 | ± | 1.13 | 5 | 50.78 | ± | 2.17 | 5 | 50.18 | **±** | 0.99 | 4 | 50.94 | ± | 1.54 | 5 | 51.12 | | ± | 1.94 | 5 | 51.62 | | ± | 2.54 | 5 | 51.56 | | ± | 1.41 | 5 | 51.66 | | ± | 1.38 | 5 | 51.56 | | ± | 1.32 | 5 | 51.22 | | ± | 1.97 | 5 |
| **MCH(pg)** | 15.90 | ± | 0.82 | 5 | 15.34 | ± | 0.31 | 5 | 15.03 | **±** | 0.25 | 4 | 15.30 | ± | 0.54 | 5 | 15.28 | | ± | 0.58 | 5 | 15.80 | | ± | 0.64 | 5 | 15.60 | | ± | 0.25 | 5 | 16.10 | | ± | 0.36 | 5 | 15.76 | | ± | 0.70 | 5 | 15.64 | | ± | 0.70 | 5 |
| **MCHC(g/dL)** | 30.94 | ± | 1.14 | 5 | 30.20 | ± | 0.70 | 5 | 29.93 | **±** | 0.42 | 4 | 30.02 | ± | 0.41 | 5 | 29.88 | | ± | 0.29 | 5 | 30.62 | | ± | 0.47 | 5 | 30.32 | | ± | 0.35 | 5 | 31.14 | | ± | 0.55 | 5 | 30.58 | | ± | 0.95 | 5 | 30.58 | | ± | 0.33 | 5 |
| **Platelets(10^3^ cells/μL)** | 1104.0 | ± | 432.3 | 5 | 1527.0 | ± | 107.4 | 5 | 1485.5 | **±** | 132.26 | 4 | 1380.4 | ± | 93.0 | 5 | 1488.0 | | ± | 171.8 | 5 | 1405.6 | | ± | 225.6 | 5 | 1317.0 | | ± | 244.2 | 5 | 978.8 | | ± | 158.1 | 5 | 1210.6 | | ± | 168.2 | 5 | 1096.2 | | ± | 108.9 | 5 |
| **Neutrophils(10^3^ cells/μL)** | 0.27 | ± | 0.13 | 5 | 0.50 | ± | 0.15 | 5 | 0.31 | **±** | 0.05 | 4 | 0.28 | ± | 0.10 | 5 | 0.53 | | ± | 0.19 | 5 | 0.33 | | ± | 0.16 | 5 | 0.38 | | ± | 0.12 | 5 | 0.55 | | ± | 0.10 | 5 | 0.41 | | ± | 0.04 | 5 | 0.52 | | ± | 0.23 | 5 |
| **Neutrophils(%)** | 13.68 | ± | 2.70 | 5 | 12.46 | ± | 4.14 | 5 | 16.10 | **±** | 4.13 | 4 | 12.48 | ± | 3.72 | 5 | 20.88 | | ± | 6.31 | 5 | 17.26 | | ± | 3.01 | 5 | 7.88 | | ± | 2.03 | 5 | 12.06 | | ± | 2.10 | 5 | 12.02 | | ± | 3.83 | 5 | 17.06 | | ± | 6.16 | 5 |
| **Lymphocytes(10^3^ cells/μL)** | 1.58 | ± | 0.64 | 5 | 3.47 | ± | 1.09 | 5 | 1.58 | **±** | 0.66 | 4 | 2.09 | ± | 1.20 | 5 | 2.43 | | ± | 2.46 | 5 | 1.40 | | ± | 0.41 | 5 | 4.26 | | ± | 1.44 | 5 | 3.71 | | ± | 0.70 | 5 | 3.08 | | ± | 1.35 | 5 | 2.78 | | ± | 1.84 | 5 |
| **Lymphocytes(%)** | 79.98 | ± | 3.50 | 5 | 82.46 | ± | 4.23 | 5 | 76.98 | **±** | 7.32 | 4 | 81.96 | ± | 4.70 | 5 | 72.32 | | ± | 7.77 | 5 | 75.78 | | ± | 2.98 | 5 | 85.20 | | ± | 4.50 | 5 | 81.28 | | ± | 4.37 | 5 | 81.84 | | ± | 5.50 | 5 | 76.06 | | ± | 6.99 | 5 |
| **Monocytes(10^3^ cells/μL)** | 0.02 | ± | 0.01 | 5 | 0.05 | ± | 0.01 | 5 | 0.03 | **±** | 0.01 | 4 | 0.03 | ± | 0.01 | 5 | 0.05 | | ± | 0.02 | 5 | 0.03 | | ± | 0.02 | 5 | 0.04 | | ± | 0.02 | 5 | 0.05 | | ± | 0.03 | 5 | 0.04 | | ± | 0.02 | 5 | 0.07 | | ± | 0.05 | 5 |
| **Monocytes(%)** | 1.52 | ± | 1.39 | 5 | 1.30 | ± | 0.40 | 5 | 1.55 | **±** | 0.31 | 4 | 1.26 | ± | 0.50 | 5 | 1.82 | | ± | 0.68 | 5 | 1.76 | | ± | 0.41 | 5 | 0.94 | | ± | 0.18 | 5 | 1.22 | | ± | 0.53 | 5 | 1.20 | | ± | 0.39 | 5 | 2.26 | | ± | 0.75 | 5 |
| **Eosinophils(10^3^ cells/μL)** | 0.07 | ± | 0.03 | 5 | 0.12 | ± | 0.06 | 5 | 0.07 | **±** | 0.05 | 4 | 0.09 | ± | 0.07 | 5 | 0.10 | | ± | 0.05 | 5 | 0.08 | | ± | 0.06 | 5 | 0.21 | | ± | 0.10 | 5 | 0.18 | | ± | 0.10 | 5 | 0.13 | | ± | 0.04 | 5 | 0.12 | | ± | 0.10 | 5 |
| **Eosinophils(%)** | 3.64 | ± | 1.30 | 5 | 2.92 | ± | 1.56 | 5 | 4.03 | **±** | 3.62 | 4 | 3.18 | ± | 0.94 | 5 | 3.82 | | ± | 1.49 | 5 | 4.28 | | ± | 2.68 | 5 | 4.72 | | ± | 2.81 | 5 | 4.02 | | ± | 2.26 | 5 | 3.70 | | ± | 1.32 | 5 | 3.42 | | ± | 1.65 | 5 |
| **Basophils(10^3^ cells/μL)** | 0.00 | ± | 0.00 | 5 | 0.01 | ± | 0.00 | 5 | 0.01 | **±** | 0.01 | 4 | 0.00 | ± | 0.01 | 5 | 0.00 | | ± | 0.01 | 5 | 0.00 | | ± | 0.01 | 5 | 0.01 | | ± | 0.01 | 5 | 0.01 | | ± | 0.00 | 5 | 0.01 | | ± | 0.01 | 5 | 0.01 | | ± | 0.01 | 5 |
| **Basophils(%)** | 0.18 | ± | 0.19 | 5 | 0.24 | ± | 0.09 | 5 | 0.35 | **±** | 0.19 | 4 | 0.30 | ± | 0.23 | 5 | 0.20 | | ± | 0.12 | 5 | 0.24 | | ± | 0.18 | 5 | 0.28 | | ± | 0.19 | 5 | 0.26 | | ± | 0.18 | 5 | 0.34 | | ± | 0.17 | 5 | 0.24 | | ± | 0.13 | 5 |
| **Reticulocyte(10^9^ cells/μL)** | 360.2 | ± | 27.9 | 5 | 347.2 | ± | 45.2 | 5 | 456.03 | **±** | 83.38 | 4 | 407.1 | ± | 32.2 | 5 | 410.4 | | ± | 73.7 | 5 | 332.7 | | ± | 178.7 | 5 | 379.3 | | ± | 137.7 | 5 | 373.2 | | ± | 168.5 | 5 | 300.6 | | ± | 154.4 | 5 | 493.0 | | ± | 101.2 | 5 |
| **Reticulocyte(%)** | 4.17 | ± | 0.32 | 5 | 4.10 | ± | 0.56 | 5 | 5.33 | **±** | 0.86 | 4 | 4.76 | ± | 0.44 | 5 | 4.85 | | ± | 0.80 | 5 | 3.77 | | ± | 2.01 | 5 | 4.50 | | ± | 1.67 | 5 | 4.58 | | ± | 1.96 | 5 | 3.57 | | ± | 1.89 | 5 | 5.96 | | ± | 1.13 | 5 |
| S.D., Standard Deviation | | | | | | | | | | | | | | | | | | | | | | | | | | | | | | | | | | | | | | | | | | | | | | |
| N, Number of Animals | | | | | | | | | | | | | | | | | | | | | | | | | | | | | | | | | | | | | | | | | | | | | | |
| ^1^, No statistical significance was observed | | | | | | | | | | | | | | | | | | | | | | | | | | | | | | | | | | | | | | | | | | | | | | |

**Supplementary data 7. Group summary of hematology in study #1 (14 day post second injection)** The levels of specified parameters were evaluated by CBC on necropsy day (14 dpsi). The data were presented with Mean ± SD (n=5), and the differences between the negative control and each test group were examined using the Kruskal-Wallis test and the Bonferroni post hoc test. No statistically significant differences were found between the D-PBS- and mRNA vaccine-injected mice.

| **<Study #1> Group Summary of Serum Blood Chemistry (14 day post second injection)^1^** | | | | | | | | | | | | | | | | | | | | | | | | | | | | | | | | | | | | | | | | |
| --- | --- | --- | --- | --- | --- | --- | --- | --- | --- | --- | --- | --- | --- | --- | --- | --- | --- | --- | --- | --- | --- | --- | --- | --- | --- | --- | --- | --- | --- | --- | --- | --- | --- | --- | --- | --- | --- | --- | --- | --- |
| **Sex** | **Male** | | | | | | | | | | | | | | | | | | | | **Female** | | | | | | | | | | | | | | | | | | | |
| **Test Item** | **D-PBS** | | | | **CUK3-1/LNP-A** | | | | **CUK3-1/LNP-B** | | | | **CUK3-1/LNP-C** | | | | **CUK3-1/LNP-D** | | | | **D-PBS** | | | | **CUK3-1/LNP-A** | | | | **CUK3-1/LNP-B** | | | | **CUK3-1/LNP-C** | | | | **CUK3-1/LNP-D** | | | |
| **mRNA Dosage (μg/head)** | **0** | | | | **50** | | | | **50** | | | | **50** | | | | **50** | | | | **0** | | | | **50** | | | | **50** | | | | **50** | | | | **50** | | | |
| **Parameter** | **MEAN** |  | **S.D.** | **N** | **MEAN** |  | **S.D.** | **N** | **MEAN** |  | **S.D.** | **N** | **MEAN** |  | **S.D.** | **N** | **MEAN** |  | **S.D.** | **N** | **MEAN** |  | **S.D.** | **N** | **MEAN** |  | **S.D.** | **N** | **MEAN** |  | **S.D.** | **N** | **MEAN** |  | **S.D.** | **N** | **MEAN** |  | **S.D.** | **N** |
| **Calcium(mg/dL)** | 8.64 | **±** | 0.19 | 5 | 8.74 | **±** | 0.25 | 5 | 8.84 | **±** | 0.18 | 5 | 8.64 | **±** | 0.09 | 5 | 8.66 | **±** | 0.17 | 5 | 8.60 | **±** | 0.16 | 5 | 8.64 | **±** | 0.34 | 5 | 8.52 | **±** | 0.16 | 5 | 8.66 | **±** | 0.36 | 5 | 8.70 | **±** | 0.28 | 5 |
| **Inorganic Phosphorus(mg/dL)** | 6.52 | **±** | 0.38 | 5 | 7.66 | **±** | 1.28 | 5 | 7.20 | **±** | 0.82 | 5 | 6.88 | **±** | 0.39 | 5 | 7.78 | **±** | 0.79 | 5 | 6.34 | **±** | 0.84 | 5 | 6.40 | **±** | 0.92 | 5 | 6.70 | **±** | 0.42 | 5 | 6.58 | **±** | 0.74 | 5 | 6.34 | **±** | 1.03 | 5 |
| **Glucose(mg/L)** | 93.00 | **±** | 13.45 | 5 | 99.20 | **±** | 24.26 | 5 | 124.60 | **±** | 29.83 | 5 | 110.60 | **±** | 22.55 | 5 | 122.40 | **±** | 26.18 | 5 | 177.80 | **±** | 25.99 | 5 | 189.40 | **±** | 20.85 | 5 | 175.40 | **±** | 20.46 | 5 | 169.80 | **±** | 15.58 | 5 | 187.40 | **±** | 20.23 | 5 |
| **Blood Urea Nitrogen(mg/dL)** | 24.32 | **±** | 1.44 | 5 | 21.86 | **±** | 4.66 | 5 | 25.06 | **±** | 2.80 | 5 | 25.52 | **±** | 2.68 | 5 | 21.12 | **±** | 2.69 | 5 | 25.34 | **±** | 7.17 | 5 | 21.50 | **±** | 3.85 | 5 | 20.36 | **±** | 1.82 | 5 | 20.02 | **±** | 3.94 | 5 | 20.14 | **±** | 1.59 | 5 |
| **Creatinine(mg/dL)** | 0.30 | **±** | 0.02 | 5 | 0.28 | **±** | 0.02 | 5 | 0.32 | **±** | 0.04 | 5 | 0.29 | **±** | 0.02 | 5 | 0.31 | **±** | 0.03 | 5 | 0.28 | **±** | 0.05 | 5 | 0.28 | **±** | 0.02 | 5 | 0.26 | **±** | 0.02 | 5 | 0.27 | **±** | 0.03 | 5 | 0.26 | **±** | 0.04 | 5 |
| **Cholesterol(mg/dL)** | 143.0 | **±** | 14.63 | 5 | 136.40 | **±** | 18.43 | 5 | 132.20 | **±** | 24.75 | 5 | 137.20 | **±** | 37.02 | 5 | 146.00 | **±** | 19.81 | 5 | 93.80 | **±** | 12.52 | 5 | 92.40 | **±** | 13.16 | 5 | 79.60 | **±** | 19.50 | 5 | 84.00 | **±** | 20.19 | 5 | 85.40 | **±** | 23.63 | 5 |
| **Total Protein(g/dL)** | 4.88 | **±** | 0.13 | 5 | 4.86 | **±** | 0.11 | 5 | 5.16 | **±** | 0.24 | 5 | 5.06 | **±** | 0.19 | 5 | 5.10 | **±** | 0.25 | 5 | 4.70 | **±** | 0.25 | 5 | 4.82 | **±** | 0.27 | 5 | 4.96 | **±** | 0.21 | 5 | 4.92 | **±** | 0.16 | 5 | 5.10 | **±** | 0.07 | 5 |
| **Albumin(g/dL)** | 1.80 | **±** | 0.07 | 5 | 1.74 | **±** | 0.05 | 5 | 1.70 | **±** | 0.10 | 5 | 1.74 | **±** | 0.05 | 5 | 1.70 | **±** | 0.07 | 5 | 1.76 | **±** | 0.09 | 5 | 1.70 | **±** | 0.10 | 5 | 1.62 | **±** | 0.08 | 5 | 1.70 | **±** | 0.00 | 5 | 1.72 | **±** | 0.08 | 5 |
| **Total Bilirubin(mg/dL)** | 0.13 | **±** | 0.04 | 5 | 0.14 | **±** | 0.03 | 5 | 0.14 | **±** | 0.02 | 5 | 0.11 | **±** | 0.05 | 5 | 0.14 | **±** | 0.04 | 5 | 0.07 | **±** | 0.01 | 5 | 0.08 | **±** | 0.02 | 5 | 0.08 | **±** | 0.04 | 5 | 0.06 | **±** | 0.02 | 5 | 0.08 | **±** | 0.02 | 5 |
| **Alkaline Phosphatase(IU/L)** | 173.20 | **±** | 25.76 | 5 | 176.20 | **±** | 34.07 | 5 | 197.20 | **±** | 18.21 | 5 | 234.60 | **±** | 49.64 | 5 | 247.40 | **±** | 64.31 | 5 | 244.80 | **±** | 33.86 | 5 | 260.40 | **±** | 34.78 | 5 | 219.40 | **±** | 19.41 | 5 | 266.40 | **±** | 60.11 | 5 | 244.20 | **±** | 40.60 | 5 |
| **Aspartate Aminotransferase(IU/L)** | 78.60 | **±** | 20.48 | 5 | 68.40 | **±** | 11.01 | 5 | 79.00 | **±** | 9.92 | 5 | 78.80 | **±** | 8.50 | 5 | 66.80 | **±** | 7.69 | 5 | 86.00 | **±** | 15.30 | 5 | 83.40 | **±** | 22.91 | 5 | 86.20 | **±** | 26.79 | 5 | 80.00 | **±** | 12.94 | 5 | 94.60 | **±** | 34.37 | 5 |
| **Alanine Aminotransferase(IU/L)** | 28.60 | **±** | 6.15 | 5 | 30.80 | **±** | 5.76 | 5 | 29.80 | **±** | 4.21 | 5 | 35.60 | **±** | 5.13 | 5 | 28.20 | **±** | 6.10 | 5 | 33.60 | **±** | 5.98 | 5 | 32.60 | **±** | 3.58 | 5 | 32.60 | **±** | 2.88 | 5 | 36.00 | **±** | 7.48 | 5 | 35.20 | **±** | 8.38 | 5 |
| **Triglycerides(mg/dL)** | 121.80 | **±** | 56.10 | 5 | 198.40 | **±** | 78.69 | 5 | 124.00 | **±** | 53.96 | 5 | 113.80 | **±** | 34.11 | 5 | 117.60 | **±** | 48.04 | 5 | 87.40 | **±** | 55.23 | 5 | 95.40 | **±** | 20.07 | 5 | 81.40 | **±** | 41.49 | 5 | 89.00 | **±** | 34.06 | 5 | 87.80 | **±** | 55.82 | 5 |
| **HDL cholesterol(mg/dL)** | 67.20 | **±** | 9.58 | 5 | 64.80 | **±** | 4.55 | 5 | 61.00 | **±** | 7.11 | 5 | 62.80 | **±** | 10.16 | 5 | 70.60 | **±** | 7.37 | 5 | 51.60 | **±** | 5.13 | 5 | 51.00 | **±** | 5.05 | 5 | 42.80 | **±** | 8.50 | 5 | 47.00 | **±** | 7.18 | 5 | 49.80 | **±** | 10.94 | 5 |
| **LDL cholesterol(mg/dL)** | 5.80 | **±** | 1.48 | 5 | 6.00 | **±** | 1.22 | 5 | 6.60 | **±** | 2.41 | 5 | 6.20 | **±** | 3.11 | 5 | 6.00 | **±** | 2.12 | 5 | 6.40 | **±** | 0.89 | 5 | 6.20 | **±** | 0.84 | 5 | 6.00 | **±** | 1.00 | 5 | 5.20 | **±** | 1.92 | 5 | 4.80 | **±** | 1.79 | 5 |
| **Sodium(mmol/L)** | 150.20 | **±** | 1.79 | 5 | 149.80 | **±** | 1.48 | 5 | 150.00 | **±** | 0.71 | 5 | 150.20 | **±** | 1.92 | 5 | 149.40 | **±** | 0.55 | 5 | 147.20 | **±** | 1.64 | 5 | 146.80 | **±** | 0.45 | 5 | 146.00 | **±** | 1.22 | 5 | 147.40 | **±** | 1.14 | 5 | 147.80 | **±** | 0.84 | 5 |
| **Potassium(mmol/L)** | 4.74 | **±** | 0.48 | 5 | 5.04 | **±** | 0.30 | 5 | 4.96 | **±** | 0.44 | 5 | 4.84 | **±** | 0.38 | 5 | 5.08 | **±** | 0.44 | 5 | 4.60 | **±** | 0.27 | 5 | 4.14 | **±** | 0.27 | 5 | 4.34 | **±** | 0.25 | 5 | 4.46 | **±** | 0.30 | 5 | 4.34 | **±** | 0.40 | 5 |
| **Chloride(mmol/L)** | 112.40 | **±** | 2.88 | 5 | 109.60 | **±** | 1.14 | 5 | 111.40 | **±** | 1.82 | 5 | 112.40 | **±** | 2.30 | 5 | 110.20 | **±** | 1.79 | 5 | 112.20 | **±** | 1.79 | 5 | 112.20 | **±** | 1.92 | 5 | 112.00 | **±** | 2.00 | 5 | 112.80 | **±** | 1.30 | 5 | 113.60 | **±** | 1.82 | 5 |
| **Cardiac Troponin-I (pg/mL)** | 0.0 | **±** | 0.0 | 5 | 0.0 | **±** | 0.0 | 5 | 7.0 | **±** | 15.6 | 5 | 0.0 | **±** | 0.0 | 5 | 6.3 | **±** | 8.6 | 5 | 0.0 | **±** | 0.0 | 5 | 7.0 | **±** | 15.7 | 5 | 74.5 | **±** | 130.1 | 5 | 55.9 | **±** | 52.8 | 5 | 34.1 | **±** | 76.3 | 5 |
| S.D., Standard Deviation | | | | | | | | | | | | | | | | | | | | | | | | | | | | | | | | | | | | | | | | |
| N, Number of Animals | | | | | | | | | | | | | | | | | | | | | | | | | | | | | | | | | | | | | | | | |
| ^1^, No statistical significance was observed | | | | | | | | | | | | | | | | | | | | | | | | | | | | | | | | | | | | | | | | |

**Supplementary data 8. Group summary of blood chemistry in study #1 (14 day post second injection)** The parameters were analyzed via serum blood chemistry on necropsy day (14 dpsi). The data were presented with Mean ± SD (n=5), and the differences between the negative control and each test group were evaluated using the Kruskal-Wallis test and the Bonferroni post hoc test. No statistically significant differences were observed between the D-PBS- and mRNA vaccine-injected mice.

| **<Study #1> Group Summary Organ Weight (Unit: g, % of body weight, 14 day post second injection)** | | | | | | | | | | | | | | | | | | | | | | | | | | | | | | | | | | | | | | | | |
| --- | --- | --- | --- | --- | --- | --- | --- | --- | --- | --- | --- | --- | --- | --- | --- | --- | --- | --- | --- | --- | --- | --- | --- | --- | --- | --- | --- | --- | --- | --- | --- | --- | --- | --- | --- | --- | --- | --- | --- | --- |
| **Sex** | **Male** | | | | | | | | | | | | | | | | | | | | **Female** | | | | | | | | | | | | | | | | | | | |
| **Test Item** | **D-PBS** | | | | **CUK3-1/LNP-A** | | | | **CUK3-1/LNP-B** | | | | **CUK3-1/LNP-C** | | | | **CUK3-1/LNP-D** | | | | **D-PBS** | | | | **CUK3-1/LNP-A** | | | | **CUK3-1/LNP-B** | | | | **CUK3-1/LNP-C** | | | | **CUK3-1/LNP-D** | | | |
| **mRNA Dosage (μg/head)** | **0** | | | | **50** | | | | **50** | | | | **50** | | | | **50** | | | | **0** | | | | **50** | | | | **50** | | | | **50** | | | | **50** | | | |
| **Parameter** | **MEAN** |  | **S.D.** | **N** | **MEAN** |  | **S.D.** | **N** | **MEAN** |  | **S.D.** | **N** | **MEAN** |  | **S.D.** | **N** | **MEAN** |  | **S.D.** | **N** | **MEAN** |  | **S.D.** | **N** | **MEAN** |  | **S.D.** | **N** | **MEAN** |  | **S.D.** | **N** | **MEAN** |  | **S.D.** | **N** | **MEAN** |  | **S.D.** | **N** |
| **Necropsy B.W** | 36.26 | ± | 2.16 | 5 | 36.52 | ± | 1.66 | 5 | 33.28 | ± | 2.33 | 5 | 36.96 | ± | 1.76 | 5 | 34.95 | ± | 2.11 | 5 | 31.17 | ± | 2.36 | 5 | 30.40 | ± | 2.02 | 5 | 29.63 | ± | 2.34 | 5 | 29.58 | ± | 0.71 | 5 | 29.60 | ± | 1.62 | 5 |
| **Spleen** | 0.322 | ± | 0.063 | 5 | 0.291 | ± | 0.037 | 5 | ****0.447** | **±** | **0.055** | **5** | 0.400 | ± | 0.036 | 5 | *****0.486** | **±** | **0.075** | **5** | 0.383 | ± | 0.028 | 5 | 0.520 | ± | 0.120 | 5 | *****0.775** | **±** | **0.072** | **5** | ***0.562** | **±** | **0.119** | **5** | *****0.716** | **±** | **0.025** | **5** |
| **Liver** | 3.899 | ± | 0.166 | 5 | 4.199 | ± | 0.170 | 5 | 4.091 | ± | 0.137 | 5 | 4.135 | ± | 0.186 | 5 | ***4.258** | **±** | **0.204** | **5** | 4.729 | ± | 0.391 | 5 | 5.316 | ± | 0.822 | 5 | ****5.927** | **±** | **0.503** | **5** | 5.562 | ± | 0.508 | 5 | ****5.963** | **±** | **0.155** | **5** |
| **Kidney (R)** | 0.753 | ± | 0.048 | 5 | 0.751 | ± | 0.063 | 5 | 0.728 | ± | 0.081 | 5 | 0.810 | ± | 0.059 | 5 | 0.886 | ± | 0.086 | 5 | 0.546 | ± | 0.065 | 5 | 0.534 | ± | 0.066 | 5 | 0.611 | ± | 0.078 | 5 | 0.557 | ± | 0.058 | 5 | 0.605 | ± | 0.047 | 5 |
| **Kidney (L)** | 0.760 | ± | 0.053 | 5 | 0.747 | ± | 0.071 | 5 | 0.725 | ± | 0.093 | 5 | 0.778 | ± | 0.053 | 5 | 0.837 | ± | 0.091 | 5 | 0.525 | ± | 0.038 | 5 | 0.517 | ± | 0.032 | 5 | 0.601 | ± | 0.054 | 5 | 0.562 | ± | 0.057 | 5 | 0.585 | ± | 0.065 | 5 |
| **Adrenal gland (R)** | 0.0078 | ± | 0.0017 | 5 | 0.0075 | ± | 0.0021 | 5 | 0.0075 | ± | 0.0017 | 5 | 0.0064 | ± | 0.0022 | 5 | 0.0076 | ± | 0.0027 | 5 | 0.0114 | ± | 0.0022 | 5 | 0.0120 | ± | 0.0029 | 5 | 0.0142 | ± | 0.0021 | 5 | 0.0129 | ± | 0.0032 | 5 | 0.0151 | ± | 0.0031 | 5 |
| **Adrenal gland (L)** | 0.0063 | ± | 0.0017 | 5 | 0.0077 | ± | 0.0018 | 5 | 0.0075 | ± | 0.0019 | 5 | 0.0074 | ± | 0.0005 | 5 | 0.0074 | ± | 0.0017 | 5 | 0.0125 | ± | 0.0026 | 5 | 0.0144 | ± | 0.0037 | 5 | 0.0167 | ± | 0.0032 | 5 | 0.0134 | ± | 0.0023 | 5 | 0.0156 | ± | 0.0026 | 5 |
| **Prostate/Uterus** | 1.034 | ± | 0.167 | 5 | 1.247 | ± | 0.156 | 5 | 0.906 | ± | 0.090 | 5 | 1.208 | ± | 0.093 | 5 | 1.095 | ± | 0.239 | 5 | 0.641 | ± | 0.279 | 5 | 0.449 | ± | 0.139 | 5 | 0.672 | ± | 0.426 | 5 | 0.731 | ± | 0.225 | 5 | 0.468 | ± | 0.148 | 5 |
| **Testis/Ovary (R)** | 0.312 | ± | 0.055 | 5 | 0.364 | ± | 0.048 | 5 | 0.327 | ± | 0.025 | 5 | 0.310 | ± | 0.025 | 5 | 0.372 | ± | 0.044 | 5 | 0.022 | ± | 0.005 | 5 | 0.024 | ± | 0.006 | 5 | 0.032 | ± | 0.003 | 5 | 0.029 | ± | 0.006 | 5 | 0.029 | ± | 0.007 | 5 |
| **Testis/Ovary (L)** | 0.306 | ± | 0.048 | 5 | 0.345 | ± | 0.048 | 5 | 0.311 | ± | 0.021 | 5 | 0.297 | ± | 0.025 | 5 | 0.351 | ± | 0.041 | 5 | 0.021 | ± | 0.002 | 5 | 0.021 | ± | 0.006 | 5 | 0.032 | ± | 0.003 | 5 | 0.025 | ± | 0.005 | 5 | 0.025 | ± | 0.003 | 5 |
| **Thymus** | 0.130 | ± | 0.021 | 5 | 0.137 | ± | 0.030 | 5 | 0.167 | ± | 0.040 | 5 | 0.130 | ± | 0.030 | 5 | 0.154 | ± | 0.027 | 5 | 0.199 | ± | 0.021 | 5 | 0.221 | ± | 0.038 | 5 | 0.244 | ± | 0.047 | 5 | 0.234 | ± | 0.023 | 5 | 0.220 | ± | 0.043 | 5 |
| **Heart** | 0.486 | ± | 0.031 | 5 | 0.451 | ± | 0.028 | 5 | 0.467 | ± | 0.036 | 5 | 0.472 | ± | 0.032 | 5 | 0.494 | ± | 0.022 | 5 | 0.412 | ± | 0.024 | 5 | 0.416 | ± | 0.036 | 5 | 0.459 | ± | 0.031 | 5 | 0.439 | ± | 0.052 | 5 | 0.431 | ± | 0.022 | 5 |
| **Lung** | 0.546 | ± | 0.046 | 5 | 0.510 | ± | 0.048 | 5 | 0.560 | ± | 0.050 | 5 | 0.541 | ± | 0.048 | 5 | 0.566 | ± | 0.067 | 5 | 0.543 | ± | 0.034 | 5 | 0.574 | ± | 0.045 | 5 | 0.586 | ± | 0.048 | 5 | 0.561 | ± | 0.036 | 5 | 0.569 | ± | 0.036 | 5 |
| **Brain** | 1.400 | ± | 0.098 | 5 | 1.367 | ± | 0.056 | 5 | 1.414 | ± | 0.070 | 5 | 1.346 | ± | 0.094 | 5 | 1.431 | ± | 0.075 | 5 | 1.552 | ± | 0.121 | 5 | 1.555 | ± | 0.087 | 5 | 1.660 | ± | 0.152 | 5 | 1.651 | ± | 0.079 | 5 | 1.631 | ± | 0.055 | 5 |
| S.D., Standard Deviation | | | | | | | | | | | | | | | | | | | | | | | | | | | | | | | | | | | | | | | | |
| N, Number of Animals | | | | | | | | | | | | | | | | | | | | | | | | | | | | | | | | | | | | | | | | |
| *, Significantly different from the control group; p<0.05 | | | | | | | | | | | | | | | | | | | | | | | | | | | | | | | | | | | | | | | | |
| **, Significantly different from the control group; p<0.01 | | | | | | | | | | | | | | | | | | | | | | | | | | | | | | | | | | | | | | | | |
| ***, Significantly different from the control group; p<0.001 | | | | | | | | | | | | | | | | | | | | | | | | | | | | | | | | | | | | | | | | |

**Supplementary data 9. Group summary of organ weight relative to body weight in study #1 (14 day post second injection)** The absolute weight of specified organs was determined on necropsy day (14dpsi), then the absolute weight was converted to relative weight (% of body weight). The data, presented as Mean ± SD (n=5), indicated statistically significant differences between the negative control and each test group, which were analyzed by the Kruskal-Wallis test and the Bonferroni post hoc test (*p<0.05, **p<0.01, ***p<0.001).

| **<Study #1> Group Summary of Histopathological Findings (14 day post second injection)** | | | | | | | | | | |
| --- | --- | --- | --- | --- | --- | --- | --- | --- | --- | --- |
| **Sex** | **Male** | | | | | **Female** | | | | |
| **Test Item** | **D-PBS** | **CUK3-1/LNP-A** | **CUK3-1/LNP-B** | **CUK3-1/LNP-C** | **CUK3-1/LNP-D** | **D-PBS** | **CUK3-1/LNP-A** | **CUK3-1/LNP-B** | **CUK3-1/LNP-C** | **CUK3-1/LNP-D** |
| **mRNA Dosage (㎍/head)** | **0** | **50** | **50** | **50** | **50** | **0** | **50** | **50** | **50** | **50** |
| **Tissue and Observed Content** | **Incidence (a/b)** | | | | | | | | | |
| **Injection site/Quadriceps muscle** |  |  |  |  |  |  |  |  |  |  |
| No abnormalities detected | 4/5 | 5/5 | 2/5 | 3/5 | 3/5 | 5/5 | 4/5 |  | 3/5 |  |
| Infiltration, inflammatory cell (minimal) | 1/5 |  | 3/5 | 2/5 |  |  |  | 4/5 | 1/5 | 1/5 |
| Inflammation, acute (mild) |  |  |  |  |  |  |  |  |  | 1/5 |
| Inflammation, chronic (minimal) |  |  |  |  | 2/5 |  | 1/5 |  |  | 1/5 |
| Inflammation, chronic (mild) |  |  |  |  |  |  |  | 1/5 | 1/5 | 2/5 |
| Inflammation, granulomatous (mild) |  |  |  |  |  |  |  |  |  | 2/5 |
| Degeneration, myofiber (mild) |  |  |  |  |  |  |  |  |  | 1/5 |
| **Bone marrow/Femur** |  |  |  |  |  |  |  |  |  |  |
| No abnormalities detected | 5/5 | 5/5 | 5/5 | 5/5 | 4/5 | 5/5 | 5/5 | 5/5 | 5/5 | 5/5 |
| Cellularity, decreased, erythroid cell (minimal) |  |  |  |  | 1/5 |  |  |  |  |  |
| **Spleen** |  |  |  |  |  |  |  |  |  |  |
| No abnormalities detected | 5/5 | 5/5 | 5/5 | 2/5 | 2/5 | 5/5 | 5/5 | 2/5 | 3/5 | 1/5 |
| Cellularity, increased, lymphocyte, white pulp (minimal) |  |  |  | 2/5 | 2/5 |  |  |  |  |  |
| Cellularity, increased, megakaryocyte (minimal) |  |  |  | 1/5 | 1/5 |  |  | 1/5 | 1/5 | 2/5 |
| Cellularity, increased, megakaryocyte (mild) |  |  |  |  |  |  |  | 1/5 |  |  |
| Apoptosis, lymphocyte (minimal) |  |  |  |  | 1/5 |  |  |  |  |  |
| Extramedullary hematopoiesis, increased, granulopoiesis (minimal) |  |  |  |  | 1/5 |  |  | 2/5 | 2/5 | 3/5 |
| **Thymus** |  |  |  |  |  |  |  |  |  |  |
| No abnormalities detected | 5/5 | 5/5 | 5/5 | 4/5 | 5/5 | 5/5 | 5/5 | 5/5 | 5/5 | 4/5 |
| Cortical atrophy (minimal) |  |  |  | 1/5 |  |  |  |  |  |  |
| Tingible body macrophage, increased (minimal) |  |  |  |  |  |  |  |  |  | 1/5 |
| a, Number of observed animals. |  |  |  |  |  |  |  |  |  |  |
| b, Number of total animals |  |  |  |  |  |  |  |  |  |  |

**Supplementary data 10. Group summary of histopathological findings in study #1 (14 day post second injection)** mRNA vaccine candidatesinduced histopathological changes in major organs (Liver, Lung, Kidney, Adrenal gland, Heart, Femur bone marrow, Quadriceps muscle, Testis/Ovary, Thymus, and Spleen) were analyzed using H&E stain. Histopathological changes-confirmed organs and observed contents were presented as the incidence. Results of 14 dpsi.


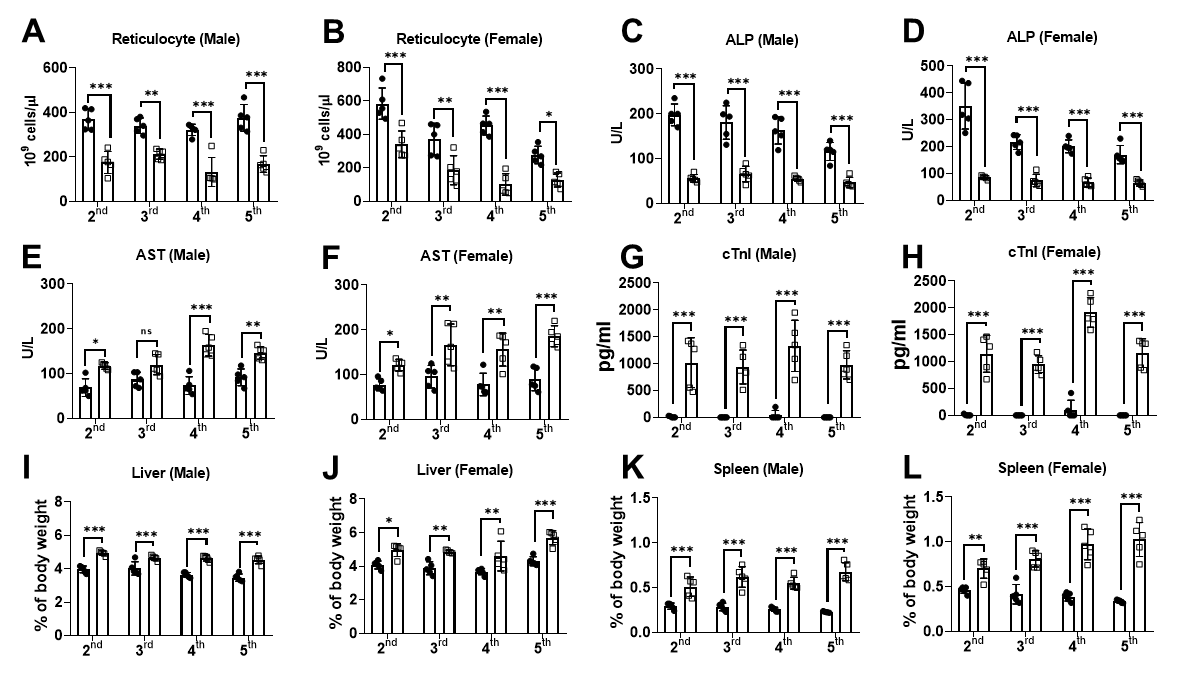


**Supplementary data 11. Group summary of hematology, blood chemistry, organ weight relative to body weight in study #2, repeated doses administration.** (A-L) The levels of the indicated parameters were analyzed on necropsy day (2 days post final injection). The data were presented as Mean ± SD (n=5), and the statistically significant differences between the negative control and each test group were analyzed using the Kruskal-Wallis test followed by the Bonferroni post hoc test (*p<0.05, **p<0.01, ***p<0.001).

| **<Study #2> Group Summary of Histopathological Findings (2 day post final injection)** | | | | | | | | | | | | |
| --- | --- | --- | --- | --- | --- | --- | --- | --- | --- | --- | --- | --- |
| **Sex** | **Male** | | | | | | **Female** | | | | | |
| **Test Item** | **3 doses** | | **4 doses** | | **5 doses** | | **3 doses** | | **4 doses** | | **5 doses** | |
|  | **D-PBS** | **CUK3-1/LNP-B** | **Vehicle** | **CUK3-1/LNP-B** | **Vehicle** | **CUK3-1/LNP-B** | **D-PBS** | **CUK3-1/LNP-B** | **Vehicle** | **CUK3-1/LNP-B** | **Vehicle** | **CUK3-1/LNP-B** |
| **mRNA Dosage (㎍/head)** | **0** | **50** | **0** | **50** | **0** | **50** | **0** | **50** | **0** | **50** | **0** | **50** |
| **Tissue and Observed Content** | **Incidence (a/b)** | | | | | | | | | | | |
| **Liver** |  |  |  |  |  |  |  |  |  |  |  |  |
| No abnormalities detected | 4/5 | 5/5 | 4/5 | 2/5 | 4/5 | 1/5 | 2/5 | 3/5 | 3/5 | 3/5 | 3/5 |  |
| Infiltration, inflammatory cell (minimal) | 1/5 |  | 1/5 | 3/5 | 1/5 | 4/5 | 3/5 | 2/5 | 2/5 | 2/5 | 2/5 | 4/5 |
| Extramedullary hematopoiesis (minimal) |  |  |  |  |  |  |  |  |  |  |  | 3/5 |
| Mineralization, hepatocyte (minimal) |  |  |  |  |  |  |  |  |  |  |  |  |
| **Injection site/Quadriceps muscle** |  |  |  |  |  |  |  |  |  |  |  |  |
| No abnormalities detected | 5/5 | 1/5 | 4/5 |  | 5/5 |  | 5/5 |  | 5/5 |  | 3/4* |  |
| Infiltration, inflammatory cell (minimal) |  |  |  |  |  |  |  |  |  |  | 1/4* |  |
| Inflammation, acute (minimal) |  | 1/5 | 1/5 |  |  |  |  |  |  | 1/5 |  |  |
| Inflammation, acute (mild) |  | 2/5 |  | 5/5 |  | 1/5 |  | 3/4* |  | 1/5 |  | 2/5 |
| Inflammation, acute (moderate) |  | 1/5 |  |  |  | 4/5 |  | 1/4* |  | 3/5 |  | 3/5 |
| Degeneration, myofiber (minimal) |  |  |  | 2/5 |  |  |  |  |  |  |  |  |
| Necrosis, myofiber (minimal) |  | 1/5 |  |  |  | 2/5 |  |  |  | 1/5 |  | 1/5 |
| Degeneration & necrosis, myofiber (minimal) |  |  |  |  |  | 2/5 |  |  |  |  |  | 1/5 |
| Degeneration & necrosis, myofiber (moderate) |  |  |  |  |  |  |  |  |  |  |  | 1/5 |
| **Bone marrow/Femur** |  |  |  |  |  |  |  |  |  |  |  |  |
| No abnormalities detected | 5/5 | 1/5 | 4/5 |  | 5/5 |  | 4/5 |  | 5/5 |  | 5/5 |  |
| Cellularity, decreased, erythroid cell (minimal) |  |  | 1/5 |  |  |  |  |  |  |  |  |  |
| Cellularity, decreased, erythroid cell (mild) |  | 3/5 |  | 1/5 |  |  |  |  |  |  |  | 2/5 |
| Cellularity, decreased, erythroid cell (moderate) |  | 1/5 |  | 2/5 |  | 1/5 |  |  |  | 4/5 |  | 3/5 |
| Cellularity, decreased, erythroid cell (severe) |  |  |  | 2/5 |  | 4/5 |  | 4/4* |  | 1/5 |  |  |
| Inflammation, granulomatous (minimal) |  |  |  |  |  |  | 1/5 |  |  | 2/5 |  |  |
| **Spleen** |  |  |  |  |  |  |  |  |  |  |  |  |
| No abnormalities detected | 4/5 |  | 4/5 |  | 5/5 |  | 4/5 |  | 2/5 |  | 3/5 |  |
| Cellularity, increased, lymphocyte, white pulp (minimal) | 1/5 | 2/5 |  | 4/5 |  | 3/5 |  | 2/5 |  | 4/5 |  | 1/5 |
| Cellularity, increased, lymphocyte, white pulp (mild) |  | 1/5 |  |  |  |  |  |  |  | 1/5 |  | 2/5 |
| Infiltration, neutrophil, red pulp (minimal) |  | 5/5 |  | 4/5 |  | 3/5 |  | 2/5 |  |  |  | 2/5 |
| Infiltration, neutrophil, red pulp (mild) |  |  |  |  |  | 2/5 |  |  |  |  |  |  |
| Cellularity, decreased, erythroid cell, red pulp (minimal) |  | 1/5 | 1/5 | 2/5 |  | 2/5 | 1/5 | 1/5 | 3/5 |  | 1/5 | 2/5 |
| Cellularity, decreased, erythroid cell, red pulp (mild) |  |  |  | 2/5 |  | 2/5 |  | 3/5 |  | 4/5 | 1/5 | 3/5 |
| Cellularity, increased, megakaryocyte (minimal) |  | 5/5 |  | 2/5 |  | 2/5 |  | 3/5 |  | 1/5 |  | 3/5 |
| Cellularity, increased, megakaryocyte (mild) |  |  |  | 3/5 |  | 3/5 |  |  |  | 4/5 |  | 2/5 |
| Extramedullary hematopoiesis, increased, granulopoiesis (minimal) |  | 2/5 |  | 2/5 |  | 3/5 |  | 4/5 |  | 2/5 |  | 1/5 |
| Extramedullary hematopoiesis, increased, granulopoiesis (mild) |  |  |  |  |  |  |  |  |  | 2/5 |  | 3/5 |
| **Thymus** |  |  |  |  |  |  |  |  |  |  |  |  |
| No abnormalities detected | 5/5 | 3/5 | 5/5 | 1/5 | 5/5 | 1/5 | 5/5 | 2/5 | 5/5 |  | 5/5 |  |
| Cortical atrophy (minimal) |  | 1/5 |  | 1/5 |  | 3/5 |  | 1/5 |  |  |  | 2/5 |
| Cortical atrophy (mild) |  | 1/5 |  | 3/5 |  | 1/5 |  | 1/5 |  | 1/4* |  | 2/5 |
| Cortical atrophy (moderate) |  |  |  |  |  |  |  |  |  | 2/4* |  |  |
| Cortical atrophy (severe) |  |  |  |  |  |  |  |  |  | 1/4* |  | 1/5 |
| Tingible body macrophage, increased (minimal) |  | 1/5 |  | 3/5 |  | 2/5 |  | 3/5 |  | 3/4* |  | 3/5 |
| Tingible body macrophage, increased (mild) |  | 1/5 |  | 1/5 |  | 1/5 |  |  |  | 1/4* |  | 1/5 |
| a, Number of observed animals. | | | | | | | | | | | | |
| b, Number of total animals | | | | | | | | | | | | |
| ^*^, The tissue of one individual was lost in the sample preparation process | | | | | | | | | | | | |

**Supplementary data 12. Group summary of histopathological findings at 2 days post final injection in study #2, repeated doses administration** mRNA vaccine candidatesinduced histopathological changes in major organs (Liver, Lung, Kidney, Adrenal gland, Heart, Femur bone marrow, Quadriceps muscle, Testis/Ovary, Thymus, and Spleen) were analyzed using H&E stain. Histopathologically confirmed organs and observed contents were presented as the incidence.

| **<Study #3> Group Summary of Histopathological Findings (2 day post second injection)** | | | | | |
| --- | --- | --- | --- | --- | --- |
| **Sex** | **Male** | | | | |
| **Test Item (Injection Route)** | **D-PBS** | **CUK3-1/LNP-B**  **(I.V)** | **CUK3-1/LNP-B (I.M)** | **CUK3-1/LNP-C**  **(I.V)** | **CUK3-1/LNP-C**  **(I.M)** |
| **mRNA Dosage (㎍/head)** | **0** | **50** | **50** | **50** | **50** |
| **Tissue and Observed Content** | **Incidence (a/b)** | | | | |
| **Liver** |  |  |  |  |  |
| No abnormalities detected | 2/5 |  |  |  | 4/5 |
| Infiltration, inflammatory cell (minimal) | 3/5 | 5/5 | 3/5 | 5/5 | 1/5 |
| Extramedullary hematopoiesis (minimal) |  | 2/5 | 2/5 |  |  |
| **Injection site / Quadriceps muscle** |  |  |  |  |  |
| No abnormalities detected | 5/5 | 5/5 |  | 5/5 |  |
| Inflammation, acute (minimal) |  |  | 1/5 |  | 1/5 |
| Inflammation, acute (mild) |  |  | 4/5 |  | 4/5 |
| **Injection site / Tail** |  |  |  |  |  |
| No abnormalities detected | 4/4* | 1/4* | 5/5 | 3/5 | 5/5 |
| Infiltration, inflammatory cell, perivascular (minimal) |  | 2/4* |  | 2/5 |  |
| Inflammation, acute (minimal) |  | 1/4* |  |  |  |
| **Bone marrow/Femur** |  |  |  |  |  |
| No abnormalities detected | 5/5 |  |  | 2/5 |  |
| Cellularity, decreased, erythroid cell (minimal) |  | 3/5 |  | 2/5 | 1/5 |
| Cellularity, decreased, erythroid cell (mild) |  | 2/5 | 4/5 | 1/5 | 3/5 |
| Cellularity, decreased, erythroid cell (moderate) |  |  | 1/5 |  | 1/5 |
| **Spleen** |  |  |  |  |  |
| No abnormalities detected | 5/5 |  |  |  | 1/5 |
| Cellularity, increased, lymphocyte, white pulp (minimal) |  | 4/5 | 2/5 | 2/5 | 1/5 |
| Cellularity, increased, lymphocyte, white pulp (mild) |  |  |  | 3/5 |  |
| Infiltration, neutrophil, red pulp (minimal) |  | 4/5 | 5/5 | 2/5 | 2/5 |
| Cellularity, decreased, erythroid cell, red pulp (minimal) |  |  | 4/5 | 1/5 | 4/5 |
| Cellularity, decreased, erythroid cell, red pulp (mild) |  | 5/5 | 1/5 | 4/5 |  |
| Cellularity, increased, megakaryocyte (minimal) |  |  | 2/5 |  | 2/5 |
| Cellularity, increased, megakaryocyte (mild) |  |  | 3/5 |  |  |
| Extramedullary hematopoiesis, increased, granulopoiesis (minimal) |  |  | 2/5 |  | 1/5 |
| Apoptosis, lymphocyte (minimal) |  | 3/5 |  | 2/5 |  |
| Apoptosis, lymphocyte (mild) |  |  |  | 3/5 |  |
| **Thymus** |  |  |  |  |  |
| No abnormalities detected | 5/5 | 1/5 |  |  | 2/5 |
| Cortical atrophy (minimal) |  |  | 2/5 | 2/5 | 1/5 |
| Cortical atrophy (mild) |  | 2/5 | 2/5 | 1/5 | 1/5 |
| Cortical atrophy (moderate) |  |  | 1/5 |  |  |
| Tingible body macrophage, increased (minimal) |  | 2/5 | 3/5 | 4/5 | 1/5 |
| Tingible body macrophage, increased (mild) |  | 2/5 | 2/5 | 1/5 | 2/5 |
| a, Number of observed animals. | | | | | |
| b, Number of total animals | | | | | |
| ^*^, The tissue of one individual was lost in the sample preparation process | | | | | |

**Supplementary data 13. Group summary of histopathological findings 2 days post second injection in study #3, administration route** Male mice were injected with D-PBS or indicated mRNA vaccine candidates via intravenous (IV) or intramuscular (IM) routes, and necropsy was performed at 2 dpsi. mRNA vaccine candidatesinduced histopathological changes in major organs (Liver, Lung, Kidney, Adrenal gland, Heart, Femur bone marrow, Quadriceps muscle, Testis/Ovary, Thymus, and Spleen) were analyzed using H&E stain. Histopathologically confirmed organs and observed contents were presented as the incidence.
